# Supplementary material for: Quantitative Temporal Viromics: An Approach to Investigate Host-Pathogen Interaction
Source: Cell. 2014 Jun 5;157(6):1460–72. doi: 10.1016/j.cell.2014.04.028 (PMC4048463; doi:10.1016/j.cell.2014.04.028)
Supplement: Data S1. Temporal Profiles of All Quantified Viral Proteins, Related to Figure 5 — (A) Temporal plots of all quantified viral proteins. (B) Profiles of proteins from Table S5A. [file mmc1.pdf]

Supplemental plots A: temporal profiles of all quantified viral proteins

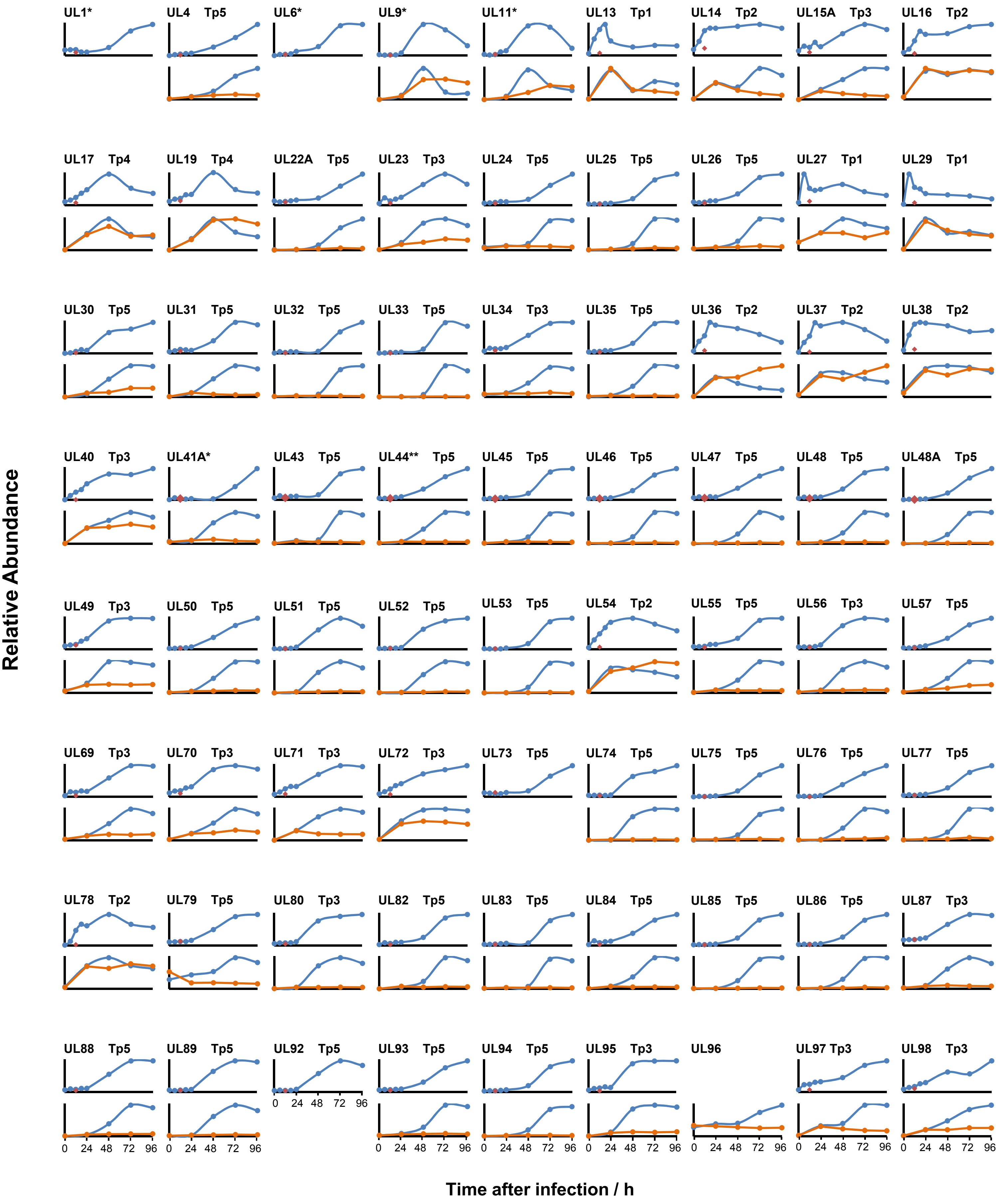

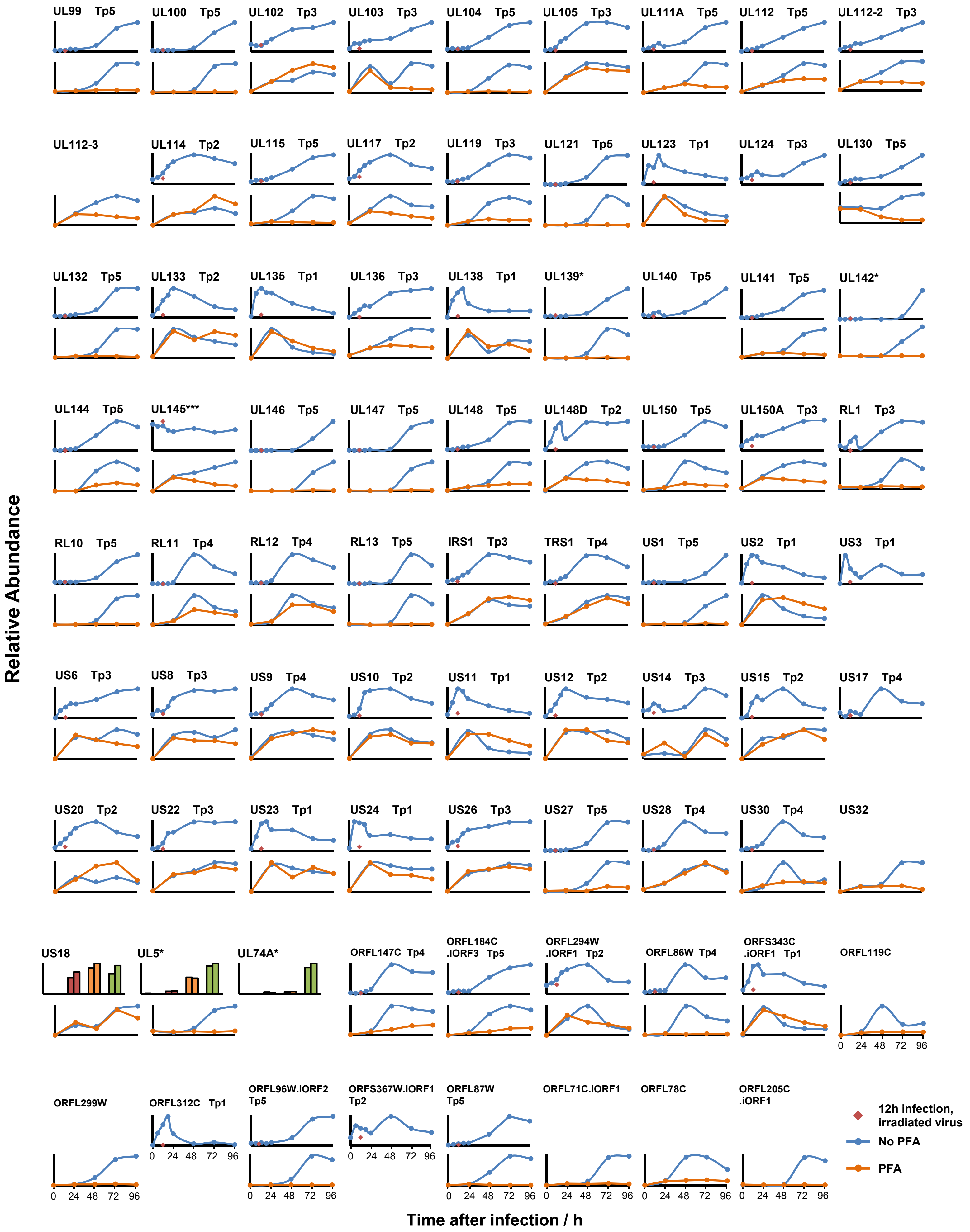

UL99 Tp5
UL100 Tp5
UL102 Tp3
UL103 Tp3
UL104 Tp5
UL105 Tp3
UL111A Tp5
UL112 Tp5
UL112-2 Tp3
UL112-3
UL114 Tp2
UL115 Tp5
UL117 Tp2
UL119 Tp3
UL121 Tp5
UL123 Tp1
UL124 Tp3
UL130 Tp5
UL132 Tp5
UL133 Tp2
UL135 Tp1
UL136 Tp3
UL138 Tp1
UL139*
UL140 Tp5
UL141 Tp5
UL142*
UL144 Tp5
UL145***
UL146 Tp5
UL147 Tp5
UL148 Tp5
UL148D Tp2
UL150 Tp5
UL150A Tp3
RL1 Tp3
RL10 Tp5
RL11 Tp4
RL12 Tp4
RL13 Tp5
IRS1 Tp3
TRS1 Tp4
US1 Tp5
US2 Tp1
US3 Tp1
US6 Tp3
US8 Tp3
US9 Tp4
US10 Tp2
US11 Tp1
US12 Tp2
US14 Tp3
US15 Tp2
US17 Tp4
US20 Tp2
US22 Tp3
US23 Tp1
US24 Tp1
US26 Tp3
US27 Tp5
US28 Tp4
US30 Tp4
US32
US18
UL5*
UL74A*
ORFL147C Tp4
ORFL184C .iORF3 Tp5
ORFL294W .iORF1 Tp2
ORFL86W Tp4
ORFS343C .iORF1 Tp1
ORFL119C
ORFL299W
ORFL312C Tp1
ORFL96W.iORF2 Tp5
ORFS367W.iORF1 Tp2
ORFL87W Tp5
ORFL71C.iORF1
ORFL78C
ORFL205C .iORF1
0 24 48 72 96
12h infection, irradiated virus
No PFA
PFA
Relative Abundance
Time after infection / h

Supplemental plots B: profiles of proteins from Table S5A

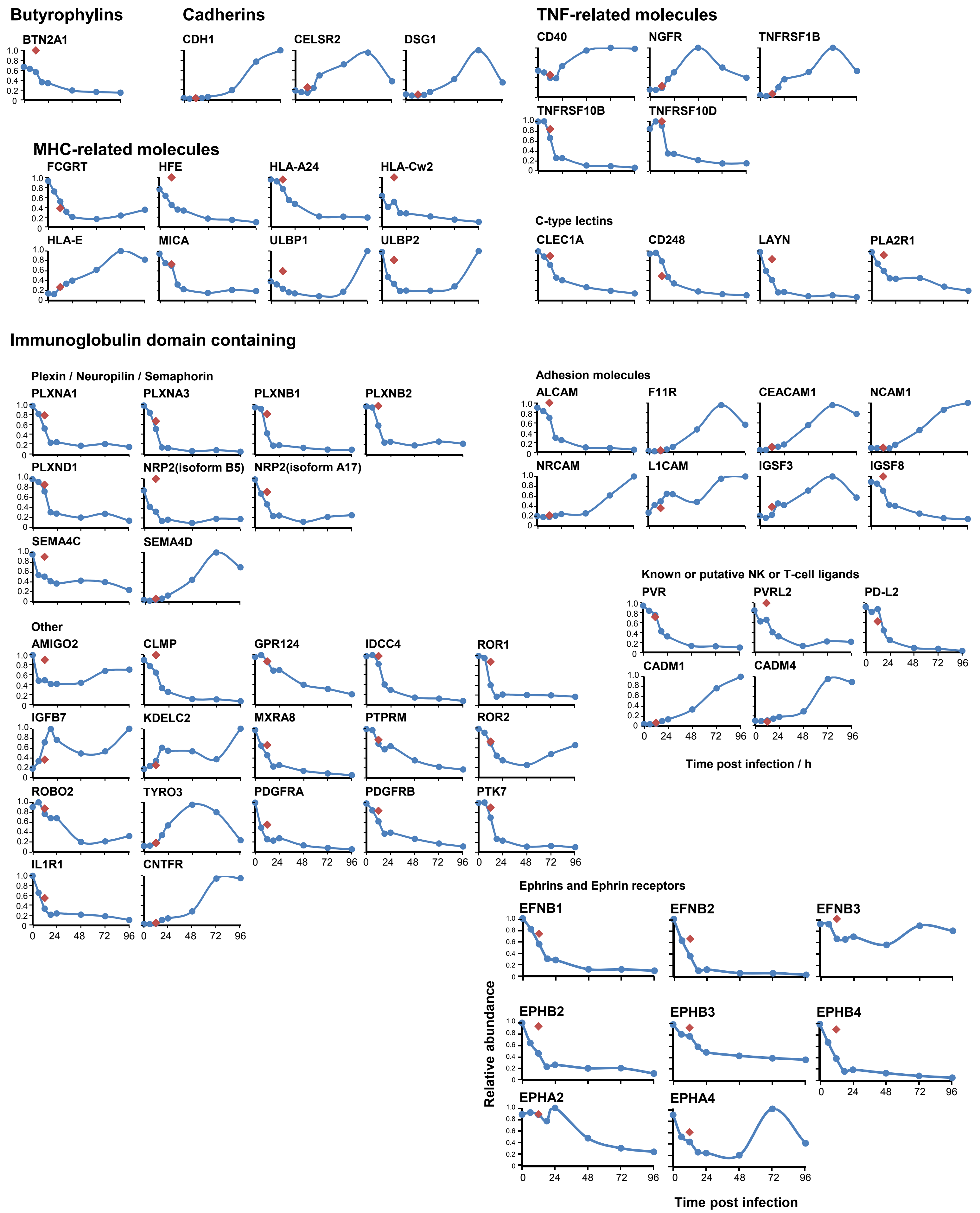

Supplemental plots A

Temporal profiles of HCMV canonical proteins and new ORFs, related to Figure 5. All 139 quantified canonical HCMV proteins and 14 new ORFs are shown. Top sets of panels - data from experiment WCL2.

*data is shown from experiment PM2, as the protein was not quantified in WCL2. Data for US18 is shown from experiment WCL1, and data for UL5 and UL74A are shown from experiment PM1 as these proteins were not quantified in experiment 2.

**UL44 has three transcription start sites utilized either from early infection onwards (sites 1, 3) or at late times only (middle start site). The same UL44 ORF is transcribed from each site, and accumulates to high levels late in infection (Leach and Mocarski, 1989). Our analysis correspondingly classified UL44 as Tp5.

***UL145 was quantified in experiment WCL2 by two peptides, one of which followed the pattern expected for a Tp3 protein, the other a likely misidentification.

Bottom sets of panels – data from experiment WCL3. The effect of the viral DNA replication inhibitor PFA can be understood by comparing protein abundance throughout infection with mock infection (where viral protein abundance relates to noise). Expression of 48/55 Tp5-class proteins was completely or almost-completely inhibited by PFA. Three UL112 isoforms were quantified: UL112, and isoforms p50 (UL112-2) and p43 (UL112-3) (Table S2). RL13 was quantified by a single peptide in both experiments WCL2 and WCL3, which was close to the N-terminus of the protein. The RL13 gene is truncated in HCMV Merlin, hence the expression kinetics we measure may not reflect the full ORF.

Supplemental plots B

Profiles are shown of all proteins from Table S5A that are not already represented in Figure 4. We quantified 3/3 ephrin B ligands, of which ephrins B1 and B2 were heavily downregulated. We quantified 3/6 Ephrin B receptors, all of which were downregulated. Ephrin receptors A2 and A4 exhibited different patterns of regulation, which were independently confirmed in experiment PM1 (Table S2).
Red diamonds – 12h after infection with irradiated HCMV. y-axes represent relative abundance.
