# Supplementary material for: Quantitative Temporal Viromics: An Approach to Investigate Host-Pathogen Interaction
Source: Cell. 2014 Jun 5;157(6):1460–72. doi: 10.1016/j.cell.2014.04.028 (PMC4048463; doi:10.1016/j.cell.2014.04.028)
Supplement: Document S1. Article plus Supplemental Information [file mmc9.pdf]

Cell

Quantitative Temporal Viromics: An Approach to Investigate Host-Pathogen Interaction

Michael P. Weekes,[1,3,4,*] Peter Tomasec,[2,4] Edward L. Huttlin,[1] Ceri A. Fielding,[2] David Nusinow,[1] Richard J. Stanton,[2] Eddie C.Y. Wang,[2] Rebecca Aicheler,[2] Isa Murrell,[2] Gavin W.G. Wilkinson,[2] Paul J. Lehner,[3] and Steven P. Gygi[1,*]
[1]Department of Cell Biology, Harvard Medical School, 240 Longwood Avenue, Boston, MA 02115, USA
[2]School of Medicine, Cardiff University, Tenovus Building, Heath Park, Cardiff CF14 4XX, UK
[3]Cambridge Institute for Medical Research, University of Cambridge, Hills Road, Cambridge CB2 0XY, UK
[4]Co-first author
*Correspondence: mpw1001@cam.ac.uk (M.P.W.), steven_gygi@hms.harvard.edu (S.P.G.)
http://dx.doi.org/10.1016/j.cell.2014.04.028
This is an open access article under the CC BY license (http://creativecommons.org/licenses/by/3.0/).

SUMMARY

A systematic quantitative analysis of temporal changes in host and viral proteins throughout the course of a productive infection could provide dynamic insights into virus-host interaction. We developed a proteomic technique called "quantitative temporal viromics" (QTV), which employs multiplexed tandem-mass-tag-based mass spectrometry. Human cytomegalovirus (HCMV) is not only an important pathogen but a paradigm of viral immune evasion. QTV detailed how HCMV orchestrates the expression of >8,000 cellular proteins, including 1,200 cell-surface proteins to manipulate signaling pathways and counterintrinsic, innate, and adaptive immune defenses. QTV predicted natural killer and T cell ligands, as well as 29 viral proteins present at the cell surface, potential therapeutic targets. Temporal profiles of >80% of HCMV canonical genes and 14 noncanonical HCMV open reading frames were defined. QTV is a powerful method that can yield important insights into viral infection and is applicable to any virus with a robust in vitro model.

INTRODUCTION

Human cytomegalovirus (HCMV) is a ubiquitous herpesvirus that persistently infects the majority of the world's population (Mocarski et al., 2013). Following primary infection, HCMV persists for the lifetime of the host under the control of a healthy immune system (Nichols et al., 2002). Reactivation from viral latency to productive infection in immunocompromised individuals, and acquisition of primary infection in utero or during transplantation can lead to serious disease (Mocarski et al., 2013). With the possibility of CMV being used as a vaccine vector (Hansen et al., 2013), a complete understanding of its ability to modulate host immunity is paramount.

During productive infection, HCMV gene expression is conventionally divided into immediate-early (IE), early (E), and late (L) phases. The *IE2* gene is primarily responsible for activating transcription of early-phase genes. By definition, early genes encode functions necessary to initiate viral DNA replication. Early-late genes (E-L) are initially transcribed at low levels and upregulated after the onset of viral DNA replication, whereas true-late genes are expressed exclusively after DNA replication and include proteins required for the assembly and morphogenesis of HCMV virions (Mocarski et al., 2013).

HCMV is a paradigm for viral immune evasion that perturbs the interferon (IFN) response (Powers et al., 2008), suppresses antigen presentation through the efficient downregulation of MHC class I (van der Wal et al., 2002), and has eight or more genes that act to suppress natural killer (NK) cell function (Wilkinson et al., 2008). Nevertheless, our understanding of how HCMV evades and modulates intrinsic immune sensors and effectors during infection remains superficial. It is not known which viral proteins are present at the cell surface, or how viral and host proteins are regulated during infection. Prior analysis of the temporal expression of HCMV genes has relied either on semiquantitative immunoblots of single viral proteins, or microarrays, which cannot capture posttranscriptional change (Ma et al., 2012). Although 604 noncanonical HCMV open reading frames (ORFs) have been identified by ribosomal footprinting (Stern-Ginossar et al., 2012), it is not yet clear how many of these ORFs encode functional polypeptides. Answering such questions has the potential to reveal mechanisms of immune evasion, cell-surface drug targets, and an improved understanding of HCMV biology.

In this paper, we describe a proteomic approach to study viral infection (quantitative temporal viromics, QTV), based on precise temporal quantitation of plasma membrane (PM) and intracellular proteins. We combined plasma membrane profiling, our recently described method for isolation of highly purified PM proteins for proteomic analysis (Weekes et al., 2012), with study of whole-cell lysates (WCL). We quantified proteins from up to ten samples using isobaric chemical tags (tandem mass tags, TMT) (McAlister et al., 2012) and MS3 mass spectrometry. This utilizes

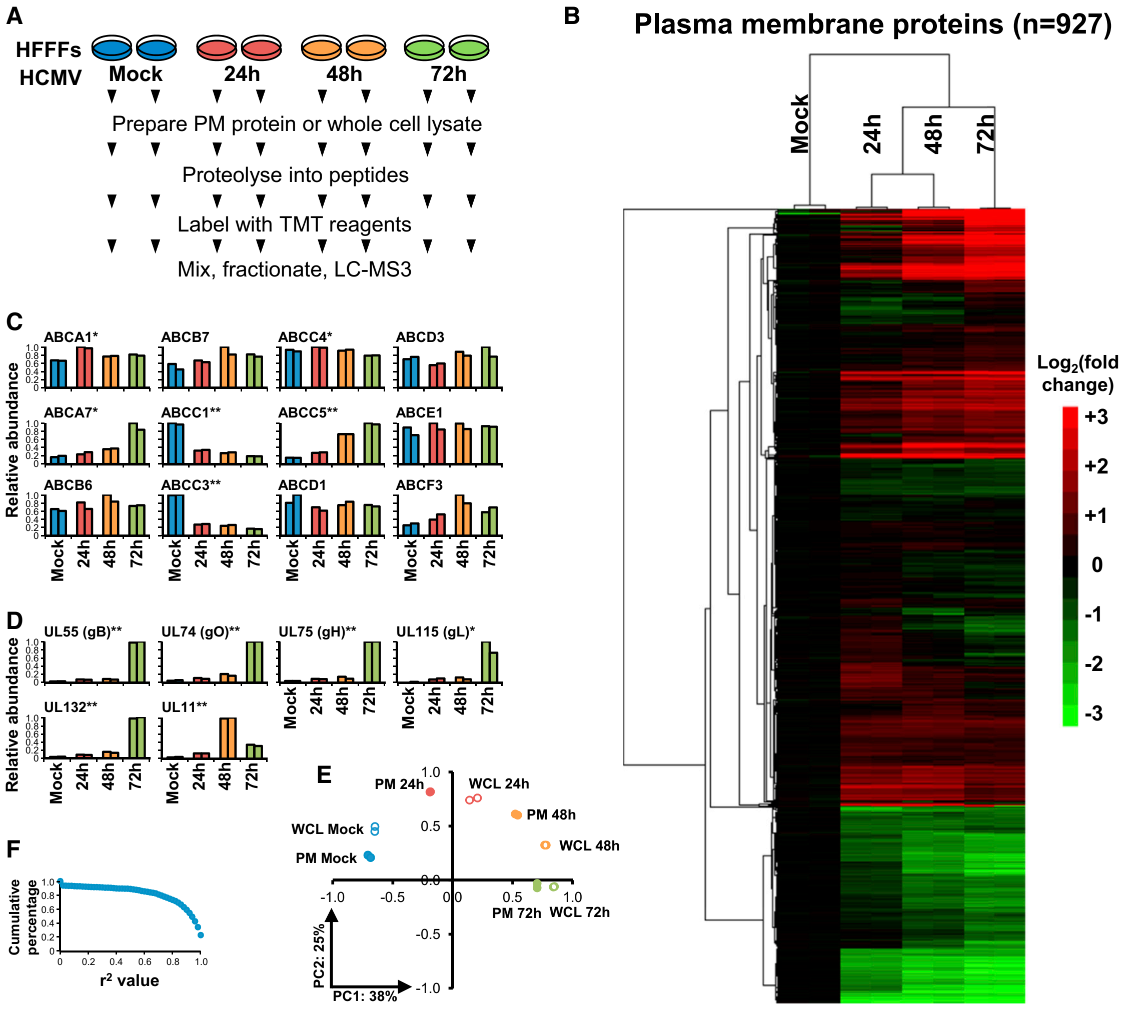

Figure 1. Temporal Plasma Membrane Profiling of HCMV-Infected Fibroblasts

(A) Workflow of experiments PM1 and WCL1.

(B) Hierarchical cluster analysis of all proteins quantified in experiment PM1 and annotated "plasma membrane," "cell surface," "extracellular," or "short GO" (Figure S1A).

(C) All ABC transporters quantified. One-way ANOVA with multiple hypothesis correction: *p < 0.005, **p < 0.0001.

(D) Quantitation of all HCMV proteins reported present at the surface of infected fibroblasts. gB, gH, gL, gO, and UL132 are virion envelope glycoproteins expressed late in infection. One-way ANOVA with multiple hypothesis correction: *p < 0.005, **p < 0.0001.

(E) Principal component analysis of all quantified proteins from experiments PM1 and WCL1 confirmed that biological replicates were highly reproducible and suggested that the major source of variability within a given experiment was duration of infection.

(F) Correlation between proteins quantified in experiments PM1 and PM2.

See also Figure S1 and Table S1.

two consecutive peptide fragmentation steps to liberate TMT reporters, minimizing interference from coisolated ions and enabling uniquely precise quantitative measurement of protein abundances at a near-global proteomic scale (Ting et al., 2011). We quantified >8,000 proteins including 1,184 cell-surface proteins and 81% of all canonical HCMV proteins over eight time points during productive infection, providing a near-complete temporal view of the host proteome and HCMV virome. Quantitative temporal viromics provides a framework for the study of any virus, enabling in-depth analysis of key aspects of viral pathogenesis.

RESULTS

Validation of Quantitative Temporal Viromics

We infected primary human fetal foreskin fibroblasts (HFFFs) with HCMV strain Merlin and initially used 8-plex TMT to quantify changes in PM protein expression. We assessed in biological duplicate three of the reference time points in productive HCMV infection and mock infection (experiment "PM1", Figure 1A). We quantified 927 PM proteins (Figure S1A available online). Surprisingly, 56% of proteins changed >2-fold and 33% >3-fold by 72 hr of infection. Replicates clustered tightly (Figure 1B).

We previously demonstrated that HCMV protein UL138 degrades the cell-surface ABC transporter Multidrug Resistance-associated Protein-1 (ABCC1) in both productive and latent infections and showed that the ABCC1-specific cytotoxic substrate Vincristine could be used therapeutically to eliminate cells latently infected with HCMV (Weekes et al., 2013). To validate findings in this present study of productive infection, we examined all quantified ABC transporters and found selective downregulation of ABCC1 as well as ABCC3 (Figure 1C). The ABCC3 drug transporter might represent an additional therapeutic target.

We further validated our results by comparison to all PM proteins that exhibit reported changes during productive HCMV infection in fibroblasts. We observed expected changes in 21/22 cellular proteins (Table S1A; Figure S1B) and detected six of six previously reported HCMV proteins at the PM. Five of these are virion envelope glycoproteins expressed late in infection (Figure 1D).

Temporal analysis of whole-cell lysates (WCLs) of HCMV-infected HFFFs enables the study of changes in expression of all proteins during infection and a comparison of the total abundance of a given protein to its expression at the PM. By analyzing WCL of HFFF infected simultaneously with PM samples, we again saw a high degree of reproducibility among biological replicates (experiment WCL1, Figures 1E and S1C) and confirmed changes in 31/35 previously reported proteins (Table S1B). We further validated our findings by comparison to a high-throughput western blot analysis of cells infected with Toledo strain of HCMV (Stanton et al., 2007) and saw a strong association between protein changes in both experiments ($p < 0.0001$, Fisher's exact test, Table S1C).

QTV to Screen for Antiviral Factors and Interferon-Induced Genes

To gain a more detailed view of changes in the host cell proteome and HCMV virome over the course of a productive infection, and to focus on events early in infection such as the IFN response, we used 10-plex TMT (McAlister et al., 2012). We analyzed productively infected HFFFs at seven time points, plus a duplicate mock infection (Figure S1D). For the tenth sample, we analyzed 12 hr infection with irradiated virus, in which viral DNA is inactivated, allowing for infection but preventing viral gene expression (Sullivan et al., 1971). Comparison of early productive infection and infection with irradiated virus allowed us to dissect the contribution of the virion to changes in the cellular proteome.

We quantified 1,184 PM proteins (experiment PM2, Figure S1E) and 7,491 cellular proteins (experiment WCL2, Figure 2A). Protein temporal profiles correlated well between repeat time courses (Figures 1F and S1F). Data from all four experiments are shown in Table S2, where the worksheet "Plots" is interactive, enabling generation of overlayed temporal graphs of PM and WCL expression of any of the human and viral proteins quantified.

Antiviral IFN effects are conferred by interferon-stimulated gene (ISG) products that target different stages of virus replication and include known herpesvirus restriction factors Viperin and IFI16 (Gariano et al., 2012). HCMV infection triggers expression of ISG, which the virus counters by impairing the IFN signaling pathway, degrading the double-stranded RNA sensor RIG-I (Retinoic acid-inducible gene I) and "repurposing" ISGs such as Tetherin to enhance rather than restrict viral replication (reviewed in Amsler et al., 2013). The temporal effects of these strategies on ISG protein expression are poorly understood as are the HCMV proteins responsible, although the immediate early proteins IE1 and IE2 and the apoptosis inhibitory protein vMIA have been implicated (Amsler et al., 2013).

One cluster of 40 proteins from experiment WCL2 (cluster A) exhibited striking upregulation at 6 and 12 hr after infection, followed by rapid return to near-basal levels (Figures 2A and 2B). Fourteen were viral proteins, and 26/40 were human, of which 85% are already known to be interferon responsive. Seventy-three percent of these proteins are additionally known to have antiviral function (Table S3A).

We therefore performed a comprehensive search of all other known interferon-induced antiviral genes, to determine which might be similarly or otherwise modulated during HCMV infection (Duggal and Emerman, 2012; Schoggins et al., 2011). Our results were consistent with the reported degradation of RIG-I and of the nuclear HCMV restriction factor Sp100 during infection (Figures 2A and S2A, respectively) (Kim et al., 2011a; Scott, 2009). Many of the tripartite motif-containing proteins (TRIMs) are interferon induced. TRIM5α can restrict HIV, although no viral factor is yet known to antagonize its expression (Rahm and Telenti, 2012). We quantified 21 TRIMs, of which TRIM 5, 16, 22, and 38 were downregulated during infection (Figure S2B). SAM Domain and HD Domain 1 (SAMHD1), zinc finger CCCH-type, antiviral 1 (ZAP/ZC3HAV1), and the novel anti-HIV factor Schlafen 11 (SLFN11) behaved similarly to members of cluster A (Figure S2A). It remains to be determined whether any of these antiviral proteins can restrict HCMV, and if reduction in their expression simply reflects diminished IFN signaling and response, or selective targeting by virus.

The relative contribution to ISG expression of active HCMV transcription (sensed, for example, by RIG-I) and viral binding to pattern recognition receptors (such as Toll-like receptor 2) is poorly understood (Paludan et al., 2011). We identified a group of 84 proteins significantly upregulated during productive infection, of which only 20% were >2-fold upregulated by irradiated virus (Figure 2C; Table S3B). The 84 proteins include 25/26 human proteins from cluster A, and 39% are known to be upregulated by IFN. Transcriptionally active HCMV may thus play an important role in ISG induction.

QTV Identifies Down- or Upregulated Signaling Pathways

The activity of a signaling pathway can be modulated either by posttranslational modification, or regulation of expression of a pathway member. Changes in protein expression can be quantified by QTV. We have shown that after an initial activation, the expression of ISG is rapidly reduced during HCMV infection, but how is this achieved? We quantified 13/15 key members of IFN induction and signaling pathways (Figure 3A, reviewed in

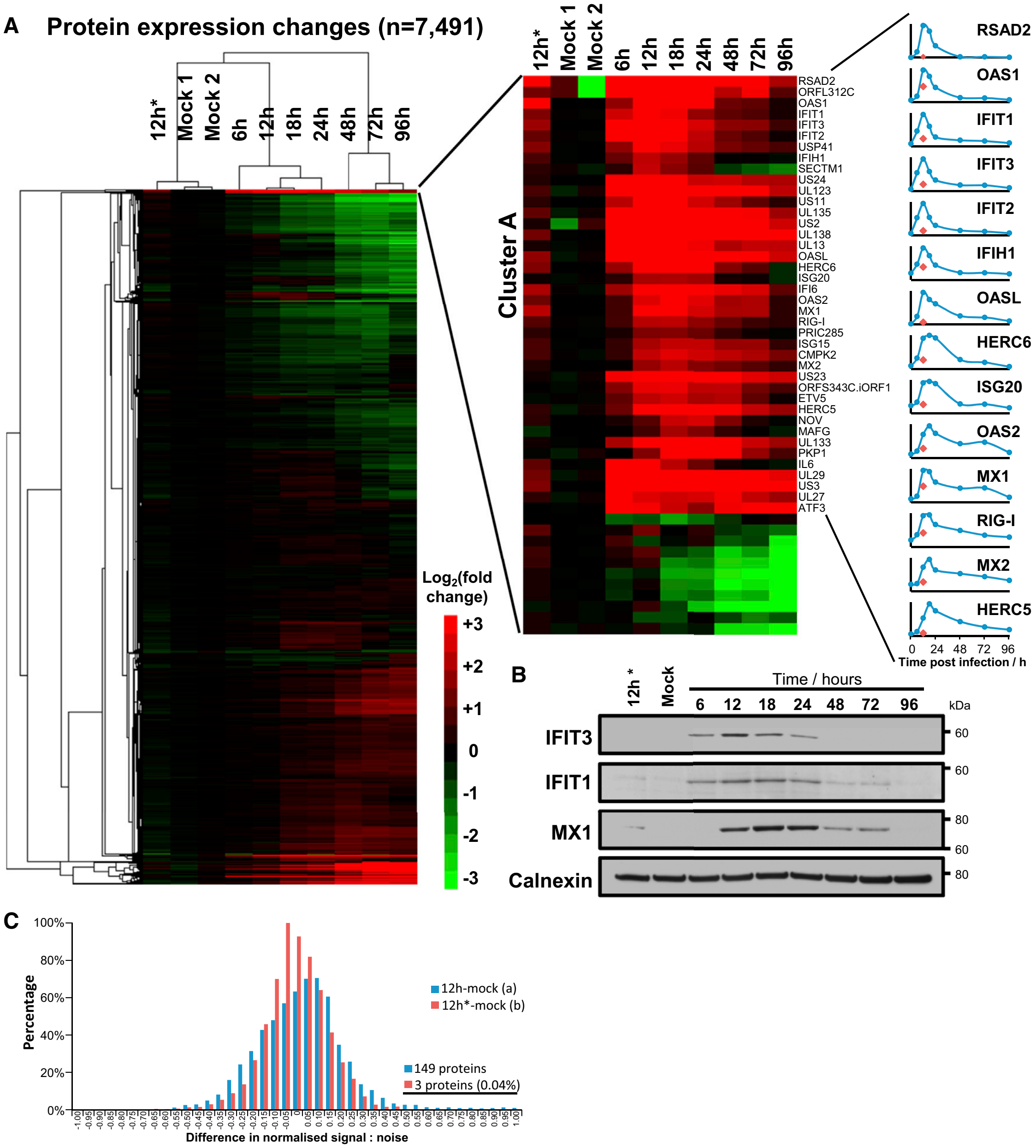

Figure 2. Temporal WCL Analysis of HCMV-Infected Fibroblasts Demonstrates Exquisite Regulation of ISGs

(A) Hierarchical cluster analysis of all proteins quantified in experiment WCL2, and enlargement of the top cluster A that included multiple IFN-induced antiviral proteins. Right panels, example temporal profiles. The y axis shows relative abundance of each protein. Red diamonds, 12 hr after infection with irradiated HCMV.

(B) Immunoblots of HFFF infected with HCMV confirm proteomic profiles.

(C) Interferon-induced proteins were more potently upregulated by productive infection than infection with irradiated HCMV. For each protein, signal:noise from the 12 hr irradiated virus or productive infection samples, and both mock samples was normalized to 1. The order of samples mock 1 and mock 2 was randomized into mock (a) and mock (b). The difference in normalized signal:noise was then calculated as indicated, and histograms were plotted. Where <0.05% of proteins were upregulated by infection with irradiated virus, 2% of proteins were upregulated by infection with live virus. These included 69 viral proteins and 84 human proteins, of which 39% are known ISGs.

See also Figure S2 and Table S3.

Amsler et al., 2013). We confirmed known effects of HCMV infection on Jak1, STAT2, and IRF9 (Le et al., 2008) and demonstrated that, apart from STAT1, expression of the final effectors in both interferon induction and response pathways were all progressively diminished during infection (Figure 3A). An effect of HCMV infection on IRF3 has not previously been reported.

The k-means method is useful to cluster proteins into a specified number of classes based on the similarity of temporal

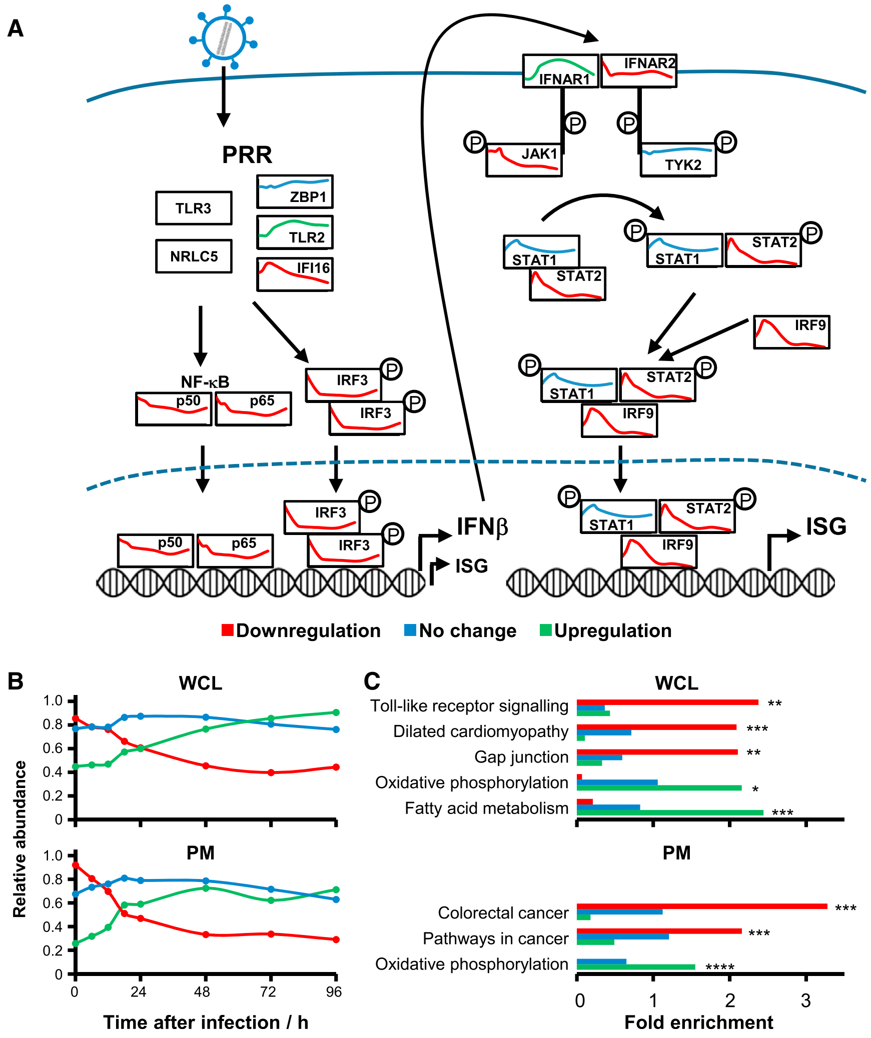

Figure 3. Modulation of Intracellular Signaling Pathways during HCMV Infection

(A) Quantitation of interferon induction and response pathways. The temporal profile of each protein is shown over 96 hr of infection, and colored red (downregulation), blue (unchanged), or green (upregulation). Data were derived from experiment WCL2 apart from IFNAR1 and IFNAR2, from PM2. Expression of certain ISGs is known to occur in the absence of IFN, in an IRF3-dependent manner. PRR, pattern recognition receptors.

(B) Average temporal profiles from 3-class k-means clustering of proteins quantified in experiments WCL2 and PM2. The three classes divided proteins into downregulated (red), unchanged (blue), or upregulated (green).

(C) Enrichment of KEGG pathways within each class was determined using DAVID software, against a background of all quantified proteins. Benjamini-Hochberg adjusted p values are shown for each indicated bar (*p < 0.00001, **p < 0.0001, ***p < 0.01, ****p < 0.05). Individual pathways are shown in Figures S3A–S3C, and pathway members are shown in Table S4.

profiles. We clustered all 7,491 proteins from experiment WCL2 and 1,184 proteins from PM2 into three classes (corresponding to upregulation, downregulation, and no change) to identify novel pathways dysregulated during HCMV infection (Figure 3B). We then applied DAVID software (Huang et al., 2009) to determine which KEGG (Kyoto Encyclopedia of Genes and Genomes) pathways were enriched in each class (Figures 3C and S3A). We made the discovery that multiple members of the TLR signaling pathway were downregulated (Figures S3A and S3B; Table S4A), suggesting that HCMV might employ a number of strategies to avoid this intrinsic immune mechanism. Fatty acid metabolism and oxidative phosphorylation were upregulated during infection (Figure 3C; Table S4A), corresponding to published literature (Koyuncu et al., 2013).

A lack of gap junction intercellular communication is common in cancer, and is thought to enable escape of homeostatic control. Gap junction alpha-1 protein (GJA1) modulates the expression of many genes involved in cell-cycle control and tumorigenesis and is degraded by HCMV IE1 and IE2 (Khan et al., 2014; Stanton et al., 2007). We confirmed GJA1 downregulation and found multiple other members of the gap junction signaling pathway similarly changed (Figure S3B; Table S4A).

The KEGG pathways "colorectal cancer" and "pathways in cancer" were enriched in the downregulated PM cluster and included Frizzled receptors FZD1, 2, 4, 6, and 7 and signaling protein WNT5a, all key members of the wnt pathway. We additionally discovered downregulation of the canonical wnt pathway coreceptors LRP5 and 6, and of noncanonical receptors ROR1, ROR2, RYK, and PTK7. Overall, 11/13 quantified wnt receptors were downmodulated (Figure S3D). Diminished transcription of wnt target genes and increased degradation of the key wnt mediator β-catenin have been reported in HCMV infection; however, the underlying mechanism is unclear (Angelova et al., 2012). Our discovery of downregulation of the majority of all wnt receptors suggests that diminished basal signaling from the cell surface may lead to increased proteasomal β-catenin degradation. Multiple effects on different cell-surface receptors suggest that the modulation of this signaling pathway may be of paramount importance to HCMV.

In order to expand our search, we performed gene set enrichment analysis (GSEA) based on the average k-means profiles for up-and downregulated WCL clusters (Figure 3B) (Subramanian et al., 2005). GSEA can increase the power to detect enrichment because it scores pathways based on similarity of protein members to a prototypic profile, as opposed to determining enrichment within a large group of proteins with broadly similar profiles. Multiple mitochondrial metabolic and biosynthetic pathways were significantly upregulated and confirmed the changes

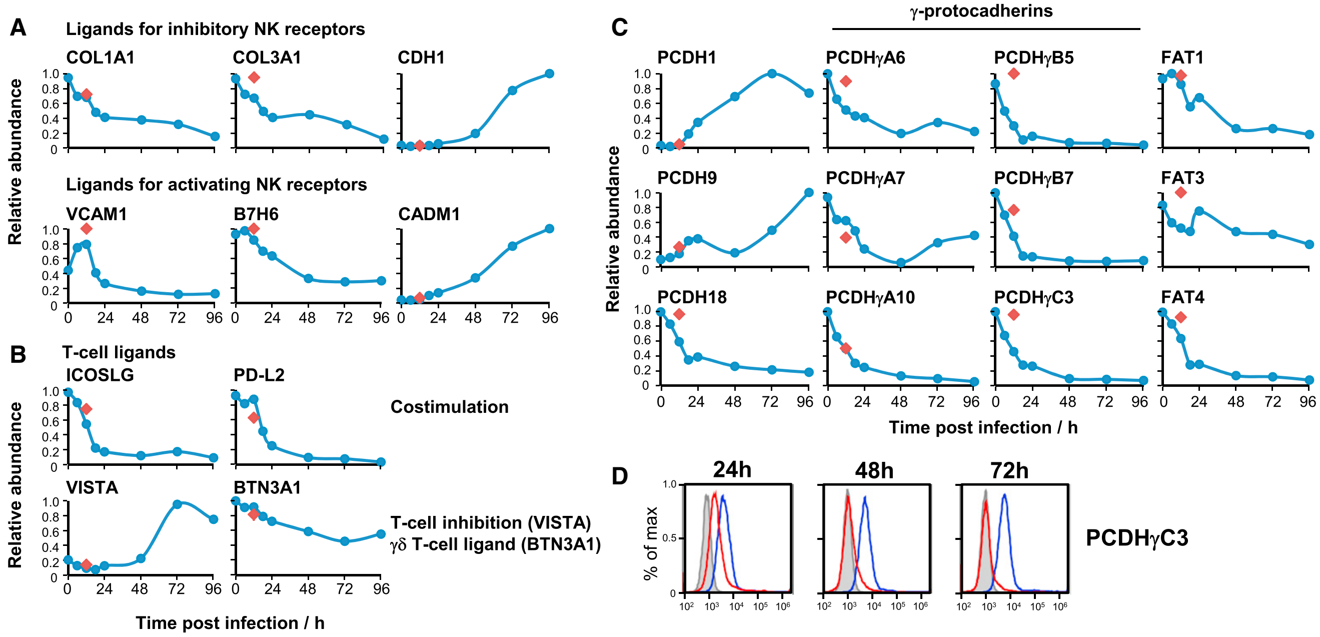

Figure 4. Temporal Changes in Known and Putative Cell-Surface Immunomodulators

(A) Temporal profiles of known NK ligands whose modulation by HCMV had not previously been recognized.

(B) Temporal profiles of T cell ligands not previously known to be modulated during infection.

(C) Temporal profiles of all quantified protocadherins.

(D) Validation of the temporal profile of PDCHγC3 by flow cytometry.

Red diamonds, 12 hr after infection with irradiated HCMV. See also Figure S4, Table S5, and Data S1.

we observed in fatty acid metabolism and oxidative phosphorylation. Transcription and export of mRNA was upregulated. Downregulated pathways included Robo receptor signaling, important in cell proliferation and motility, and ERBB and VEGFR signaling (Table S4B).

Discovery of NK and T Cell Ligands and Families of Other Cell-Surface Receptors/Ligands Modulated by HCMV Infection

We mined our data for all known NK cell ligands (Vivier et al., 2008) and discovered previously unrecognized modulation of six ligands during HCMV infection. E-cadherin (CDH1), the ligand for the inhibitory NK receptor KLRG-1 (killer cell lectin-like receptor subfamily G member 1), was dramatically upregulated during infection. Vascular cell adhesion molecule 1 (VCAM1) and B7-H6, ligands for activating NK receptors α4β1 integrin and NKp30, were downregulated (Figure 4A). Interestingly, other known ligands including collagen I (COL1A1 and COL1A2), collagen III (COL3A1), cell adhesion molecule-1 (CADM1), and poliovirus receptor-related 1 (PVRL1) were expressed in a manner that would be expected for an appropriate response to intracellular infection (Figure 4A; Table S2).

We performed a similar screen for all known αβ T cell costimulatory molecules, and γδ T cell ligands (Bonneville et al., 2010; Hubo et al., 2013). The T cell costimulators ICOSLG (inducible T cell costimulator ligand) and PD-L2 were downregulated during infection, as was butyrophilin subfamily 3 member A1 (BTN3A1), recently shown to present phosphoantigens to Vγ9Vδ2$^+$ T cells (Vavassori et al., 2013). V-domain Ig suppressor of T cell activation (VISTA, C10Orf54), a novel B7 family inhibitory ligand (Ceeraz et al., 2013) was upregulated late in infection (Figure 4B).

NK and T cell ligands belong to a few key protein families, including cadherins, C-type lectins, immunoglobulin, TNF receptor, and major histocompatibility-complex-related molecules (Vivier et al., 2008). In order to discover immunomodulating proteins, we added InterPro functional domain annotations to data from experiments PM1 and PM2 (Hunter et al., 2012) and reasoned that modulation of a ligand during HCMV infection may indicate biological importance. Seventy-four proteins had a relevant InterPro annotation and at least a 4-fold change compared to mock infection (Table S5A; Data S1). Eight downregulated proteins were protocadherins. Examining all quantified proteins in this family, we found that six of six γ-protocadherins were particularly potently downregulated (Figure 4C). The protocadherins may therefore represent a major set of immunomodulators.

We confirmed temporal profiles of protocadherins PCDHγC3 and FAT1 in addition to eight other cell-surface proteins by flow cytometry and immunoblot (Figures 4D and S4A, S4C, and S4E). To provide proof-of-principle validation of our functional predictions, we performed siRNA-mediated knockdown of the C-type lectin CLEC1A (Table S5A), which is downregulated 7-fold by HCMV and is a potential NK ligand by homology to CLEC2D. Polyclonal NK-cells from three of three independent donors demonstrated reduced degranulation to autologous

targets upon knockdown of CLEC1A suggesting that this molecule may be a novel activating NK ligand (Figure S4D). We observed similar results for the protocadherin FAT1, providing initial confirmatory evidence that members of this family may indeed be involved in immunoregulation (Figure S4E). CEACAM-1 (immunoglobulin family) was upregulated 20-fold by HCMV infection and may have roles in T cell regulation. Using a blocking antibody, we demonstrated increased degranulation of $CD8^+$ T cells specific for an HCMV HLA-A2 restricted peptide epitope suggesting that upregulation of this molecule in infected cells may inhibit cytotoxic T cell-mediated lysis (Figure S4F).

There is increasing evidence for a substantial role of plexin-semaphorin signaling in the immune system (Takamatsu and Kumanogoh, 2012). For example, secreted class III semapohrins bind plexins A and D1 to regulate migration of dendritic cells to secondary lymphoid organs. Plexin B2 interacts with membrane-bound semaphorin 4D to promote epidermal $\gamma\delta$ T cell activation (Witherden et al., 2012). HCMV infection substantially downregulated five of the nine plexins: A1, A3, B1, B2, and D1. Intriguingly, neuropilin 2, a plexin coreceptor was also rapidly downregulated. Semaphorin 4D was dramatically upregulated and 4C downregulated, suggesting that viral interaction with these ligands is complex (Table S5A; Data S1).

To determine which protein domains and biological processes were enriched within highly downregulated PM proteins, we used DAVID software (Huang et al., 2009). The Interpro categories "protocadherin gamma" and "immunoglobulin-like fold" were significantly enriched in addition to Gene Ontology (GO) biological processes "regulation of leukocyte activation" and "positive regulation of cell motion," suggesting a negative effect of HCMV on cell motility. DAVID analysis also revealed families of downregulated proteins, including six G-protein-coupled receptors from the rhodopsin-like superfamily (Table S5B).

Within the category "regulation of leukocyte activation," ephrin B1 was downregulated, and we therefore examined all ephrins and their receptors. We observed striking downregulation of ephrins B1 and B2 as well as all three of their quantified B-class ephrin receptors (Data S1). Downstream effects of forward or reverse ephrin signaling include changes in cell adhesion, shape, and motility, and there is evidence for a role of ephrin B2 in murine T cell costimulation (Nikolov et al., 2013), suggesting that downregulation of this pathway may represent another mechanism of immune evasion.

Temporal Analysis of HCMV Viral Protein Expression

Most prior studies of the temporal expression of canonical HCMV proteins have employed immunoblots at only one or a few time points. Comparison between different reports has been complicated by lack of specific antibodies and study-to-study variation in the HCMV strain used, with laboratory-adapted strains AD169 and Towne (which lack a large number of ORFs as a result of extensive passage in vitro) often used in preference to isolates containing a complete genome (Ma et al., 2012). We were able to quantify the temporal expression of the majority (139/171) of the canonical HCMV proteins as well as 14 non-canonical ORFs; most of these proteins were quantified in a single experiment (Figure 5, plots for all quantified viral proteins in Data S1).

IE/E/E-L/L cascades are functionally defined by the use of metabolic inhibitors. IE transcripts accumulate in the presence of a protein synthesis inhibitor such as cycloheximide. Expression of early genes is unaffected by viral DNA synthesis inhibitors such as Phosphonoformate (PFA), whereas E-L genes are partially inhibited, and L genes completely inhibited (Chambers et al., 1999).

A complementary method of classification would be to group viral proteins according to their temporal profiles. Use of the k-means method with four classes suggested that HCMV protein profiles grouped similarly to the recognized functional cascades IE/E/E-L/L (Figure 5A). We performed k-means clustering with 2 to 14 classes and assessed the summed distance of each protein from its cluster centroid. Although this summed distance necessarily becomes smaller as more clusters are added, the rate of decline decreases with each added group, eventually settling at a fairly constant rate of decline that reflects overfitting; clusters added prior to this point reflect underlying structure in the temporal protein data, whereas clusters subsequently added through overfitting are not informative. The point of inflexion fell between five and seven classes, suggesting that there are at least five distinct temporal protein (Tp) profiles of viral protein expression (Figures 5A–5D). We term these classes Tp1, Tp2, Tp3, Tp4, and Tp5. There was generally good correspondence between classical functional and protein temporal classes, for example, the known L proteins UL99, UL94, UL75, UL115, and UL32 all classified as Tp5 (Omoto and Mocarski, 2014). A cluster of 13 proteins that we call Tp4 exhibited a distinct profile to Tp3 and Tp5, with maximal expression at 48 hr and low expression at other time points (Figures 5C and 5D). Members of this cluster predominantly originated from two regions of the viral genome (Table S6A).

To directly compare classical functional and protein temporal classes, we performed WCL analysis in the presence or absence of the viral DNA replication inhibitor PFA (experiment WCL3, Figures 5C and 5D; Table S2; plots for all quantified viral proteins in Data S1). Expression of 48/55 Tp5-class proteins was completely or almost completely inhibited by PFA. Tp3 and Tp4 proteins were generally partially inhibited, with little effect on Tp1 or Tp2. Some proteins displayed more complex profiles, with enhanced expression later in infection with PFA block (Data S1).

Eight proteins appear earlier in infection than had previously been understood. UL27, UL29, UL135, UL138, US2, US11, US23, and US24 all exhibited peak expression at 6–18 hr after infection (Figure 5C). UL29 and US24 appeared particularly early, with peak expression at only 6 hr postinfection, which may correspond with their suggested ability to stimulate IE gene expression (Feng et al., 2006; Terhune et al., 2010).

We were unable to confidently resolve the profile of UL122 (IE2). UL122 and UL123 are expressed by alternative splicing of a single major immediate-early transcript. Exons 1, 2, 3, and 4 encode UL123 and exons 1, 2, 3, and 5 encode UL122; however, additional transcripts have also been detected from the region of exon 5 (Stenberg et al., 1989; Stern-Ginossar et al., 2012). We examined each peptide quantified from every exon (Figure S5A). The profiles of all peptides from exon 4 peaked at 18–24 hr, corresponding to the predicted expression of UL123

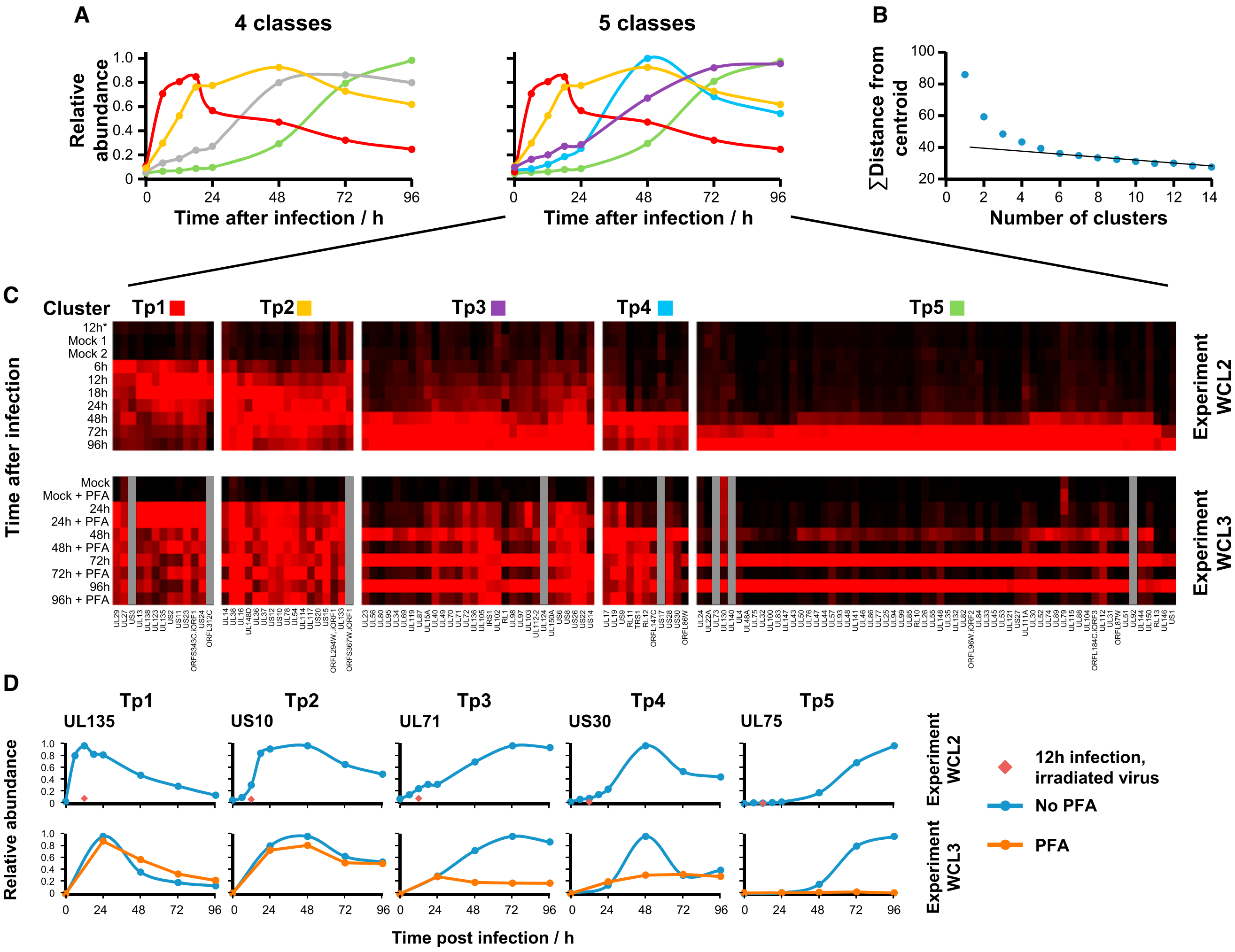

Figure 5. Definition of Temporal Classes of HCMV Gene Expression

(A) The k-means method was used to cluster all quantified HCMV proteins (experiment WCL2) into four or five classes. Shown are the average temporal profiles of each class. With four classes, proteins grouped into classes similar to the classical IE/E/E-L/L cascades. With five classes, a distinct temporal profile appeared (blue).

(B) Number of temporal classes of HCMV gene expression. The summed distance of each protein from its cluster centroid was calculated for one to 14 classes and plotted. The point of inflexion fell between five and seven classes.

(C) Top: temporal profiles of proteins in each k-means class (experiment WCL2) were subjected to hierarchical clustering by Euclidian distance. Both UL112 and isoform p50 of UL112 (UL112-2) were quantified. UL122 was excluded from clustering due to uncertainty in peptide assignment. Bottom: experiment WCL3. Viral protein profiles were further assessed in the presence or absence of the viral DNA replication inhibitor PFA. Proteins are displayed in the same order as the clusters defined in the upper panels.

(D) Temporal profiles of typical proteins from each cluster (upper panel), and the corresponding profiles in the presence or absence of PFA (lower panel).

See also Figure S5, Table S6, and Data S1.

protein. The profiles of ten exon 5 peptides corresponding to the late-expressed internal ORF, ORFL265C.iORF1 (Stern-Ginossar et al., 2012) peaked at 96 hr. A single peptide N-terminal to this ORF that is likely to originate from UL122 had a distinct profile with expression from 6 hr that peaked at 48 hr, similar to UL122 mRNA (Stern-Ginossar et al., 2012). This suggests the existence of at least two proteins arising wholly or in part from exon 5 and corresponds to the detection of an abundant late 40 kDa protein, previously detected from the same internal exon 5 ORF of AD169 strain (Stenberg et al., 1989). It is likely that ORFL265C.iORF1 protein is more abundant than UL122, effectively masking the earlier signal from most UL122 exon 5 peptides. Our quantitation of DNA polymerase processivity subunit UL44 may similarly have been complicated due to multiple transcription start sites (Data S1).

We further compared our protein-level data with recent RNA-sequencing-based temporal analysis of HCMV Merlin strain transcripts at 5, 24, and 72 hr postinfection in HFFF (Stern-Ginossar et al., 2012). We again used 5 k-means classes to group transcripts based on their temporal profiles (Figure S5C).

Because there were no intermediate mRNA time points between 5 and 24 hr, or between 24 and 72 hr, there was insufficient information to make an accurate comparison between the central three mRNA clusters and our Tp2, Tp3, or Tp4 class proteins. We therefore used mRNA data to define three classes: Tr1, Tr2–4, and Tr5 (Figure S5C). Comparison to our protein data was striking: 10/13 Tp1 proteins were also Tr1 transcripts and 49/55 Tp5 proteins were Tr5 transcripts ($p < 0.0001$, Fisher's exact test) (Table S6C; Figure S5D). Such correspondence between different studies targeting distinct classes of biomolecules suggests that the temporal classes of HCMV gene expression we define are likely to be biologically relevant.

We quantified the full temporal profiles of nine noncanonical HCMV ORFs. Four ORFs related to canonical HCMV proteins (N-terminal extension, internal ORF, C-terminal extension) and demonstrated similar temporal profiles to their canonical counterparts (Figure S5B; Data S1). Five ORFs were encoded either in different reading frames, or on the reverse strand to canonical genes (Table S6B). We additionally quantified five further noncanonical ORFs in experiment WCL3 (Data S1).

HCMV Proteins Present at the Cell Surface

Viral proteins present at the surface of an infected cell may be therapeutic antibody targets. To date, only six HCMV proteins have been demonstrated at the PM of infected fibroblasts, all late in infection, which we have confirmed (Figure 1D). Several other studies transduced individual viral genes and demonstrated protein appearance at the cell surface, which can indicate but may not reflect protein location during productive infection (Table S7).

We detected a total of 67 viral proteins in experiments PM1 and PM2. Plasma membrane profiling provides a very substantial enrichment for PM glycoproteins (up to 90% of proteins from unfractionated samples, and approximately 60% of proteins from fractionated samples have indicative Gene Ontology (GO) terms (Figure S1A) (Weekes et al., 2012). Subcellular localization of viral proteins is, however, poorly annotated, making it difficult to determine which might be non-PM contaminants, such as abundant viral tegument and nuclear proteins. We therefore developed a filtering strategy: for every GO-annotated human protein quantified in experiment PM1 or PM2 (Figure S1A), we calculated the ratio of peptides (experiments PM1 + PM2)/(experiments WCL1+WCL2). Ninety-two percent of proteins that were GO-defined non-PM had a ratio of <1.4; 88% of human proteins scoring above 1.4 were annotated as PM proteins, demonstrating the predictive value of this metric (Figure 6A). Applying this filter, we defined 29 high-confidence viral PM proteins, which included the majority of viral proteins previously identified at the surface of either infected or transduced cells, and excluded all proteins unlikely to be present at the cell surface based on their known function (Table S7).

In general, we observed a striking correlation between the PM2 and WCL2 temporal profiles of all 29 high-confidence proteins. For the subset of known virion envelope glycoproteins (Varnum et al., 2004), protein expression at the PM was significantly later than in WCL, confirmed by analysis of the same proteins from experiments PM1 and WCL1 (Figures 6B and S6). This may reflect assembly of the HCMV virion within the viral cytoplasmic assembly compartment prior to viral egress (Alwine, 2012) and demonstrates that QTV can additionally provide insights into aspects of the viral life cycle. PM appearance of UL119 was similarly delayed, suggesting that this known virion glycoprotein may be a component of the virion envelope. The sensitivity of MS3 TMT-based mass spectrometry is suggested by our apparent quantitation of binding or fusion of viral envelope with the plasma membrane; there was a small early peak in PM presence of most virion envelope glycoproteins at 6 hr of infection that disappeared by 12 hr (Figure 6B).

QTV Indicates Mechanism of Action of Viral Proteins

We have previously shown that HCMV UL141 retains the poliovirus receptor (PVR) in the endoplasmic reticulum, inhibiting cell-surface expression and preventing interaction with activating NK receptor DNAM-1. Intracellular PVR accumulates during HCMV infection. In contrast, a second DNAM-1 ligand poliovirus receptor-related 2 (PVRL2) is targeted for proteasomal degradation by UL141 acting with other HCMV gene(s) (Prod'homme et al., 2010; Tomasec et al., 2005). QTV confirmed these results; we observed rapid depletion of PVR from the plasma membrane, in contrast to its accumulation within WCL. PVRL2 was lost from both PM and WCLs (Figure 7). The WCL kinetics of UL141 expression paralleled that of PVR but were inversely related to PVRL2.

The activating receptor NKG2D is expressed on a variety of immune effector cells and recognizes major histocompatibility complex class I-related ligands MICA, MICB, and ULBP1-3. HCMV infection strongly induces transcription of MICB, ULBP1, and ULBP2 mRNA but prevents their surface expression by intracellular sequestration with HCMV UL16 (Dunn et al., 2003a). Our data were highly consistent with this literature. Interestingly, cell-surface expression of ULBP1 and -2 rose late in infection, suggesting that UL16 had become overwhelmed (Figure 7A).

We therefore examined other HCMV proteins known to target cell-surface receptors (Table S1A). The profiles of UL138 and ABCC1 were consistent with their codegradation (Weekes et al., 2013), and the profile of HLA-A reflected US2 and US11-mediated degradation (Figure 7B). Other examples are shown in Figures S7A and S7B. The temporal profiles provided by QTV are thus useful to gain mechanistic insights into known interactions between viral and host proteins.

DISCUSSION

In this study, we provide a comprehensive resource describing temporal changes in viral and host proteomes during infection with HCMV. Simultaneous study of protein expression at the cell surface and in whole-cell lysates has revealed insights into many aspects of the viral life cycle. To be able to persist in an infected individual lifelong, herpesviruses have developed a wealth of strategies to modulate innate and adaptive immunity. One benefit is that we can use HCMV infection to discover proteins important in host defense, as viral modulation of protein expression generally reflects biological imperative. We often observed modulation of multiple members of the same protein family, increasing the likelihood of biological relevance; for

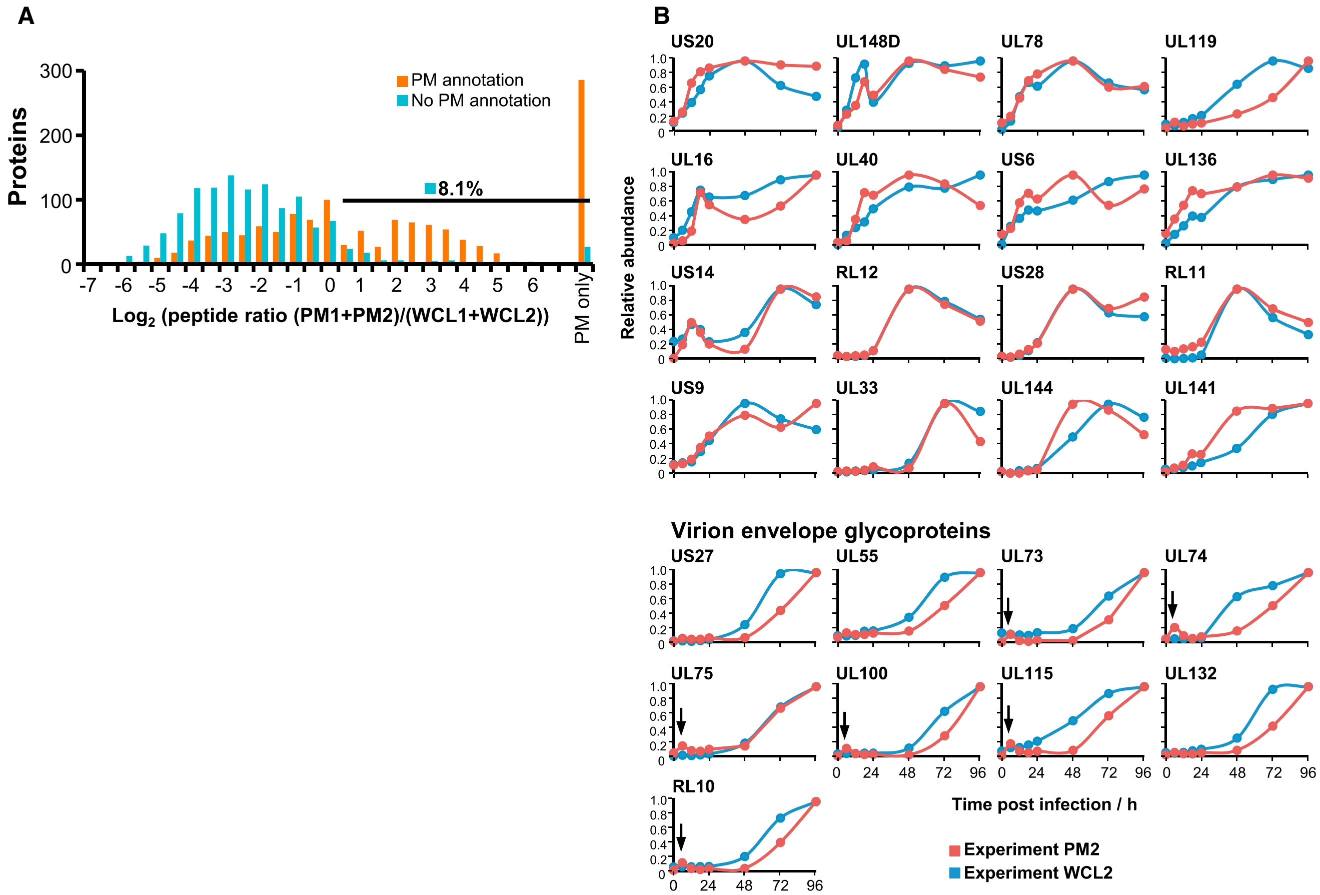

Figure 6. HCMV Proteins Quantified at the Surface of Infected Fibroblasts

(A) Histogram of peptide ratios for all GO-annotated proteins quantified in experiments PM1 or PM2. "PM only," not detected in experiments WCL1 or WCL2. "PM annotation": "plasma membrane", "cell surface," "extracellular," or "short GO."

(B) Temporal profiles of all high-confidence PM proteins (Table S6). Virion envelope glycoproteins were generally detected significantly earlier in whole-cell lysates than in plasma membrane samples. Arrows, quantitation of fusion or binding of the virion envelope and the plasma membrane.

See also Figure S6 and Table S7.

example, six of six γ-protocadherins that are downregulated during infection may be novel NK ligands in addition to protocadherin FAT1. The correspondence in temporal profiles of proteins in cluster A (Figure 2A) enables prediction of IFN-stimulated genes, which might additionally have antiviral function. Multiple signaling pathways were modulated by HCMV, which has the potential to inform not only viral interaction networks by predicting effects on pathway target genes but improves our knowledge of the metabolic changes necessary to effect viral replication (Koyuncu et al., 2013). GSEA provides a particularly useful overview of many of the pathways and receptors changing significantly in our data.

The temporal quantitation of >80% of canonical HCMV proteins in one experiment provides a significant technical advance. Our knowledge of HCMV protein profiles has so far relied on a literature complicated by the use of multiple different laboratory-adapted viral strains, different cell types, and variable infection conditions (Ma et al., 2012). Many have not previously been quantified. We provide a temporal system of classification of HCMV protein expression using the prototype clinical strain Merlin, which is complementary to and consistent with the functionally derived classical IE/E/E-L/L nomenclature. For example, expression of 87% of proteins we define as Tp5 was prevented by the viral DNA replication inhibitor PFA. These data suggest that the classical "true late" category of transcripts could be substantially expanded. Furthermore, all capsid proteins we quantified (Mocarski et al., 2013) belonged to class Tp5, whereas viral tegument proteins were more diverse: of 27 quantified, three were Tp1, 2 Tp2, 8 Tp3, and 14 Tp5, suggesting that these proteins may additionally have nonstructural roles such as regulation of gene expression and immune evasion. It remains to be seen whether Tp4 proteins have a distinct function that requires peak expression at 48 hr of infection, for example, preparation of the cell for virus release via inhibition of neutralizing antibody and cellular immunity (RL11 family), or promoting viral egress and cell-cell spread (Table S5A). Particular advantages to our temporal measurements include the ability to correlate viral and cellular protein expression, narrowing the field of viral proteins possibly

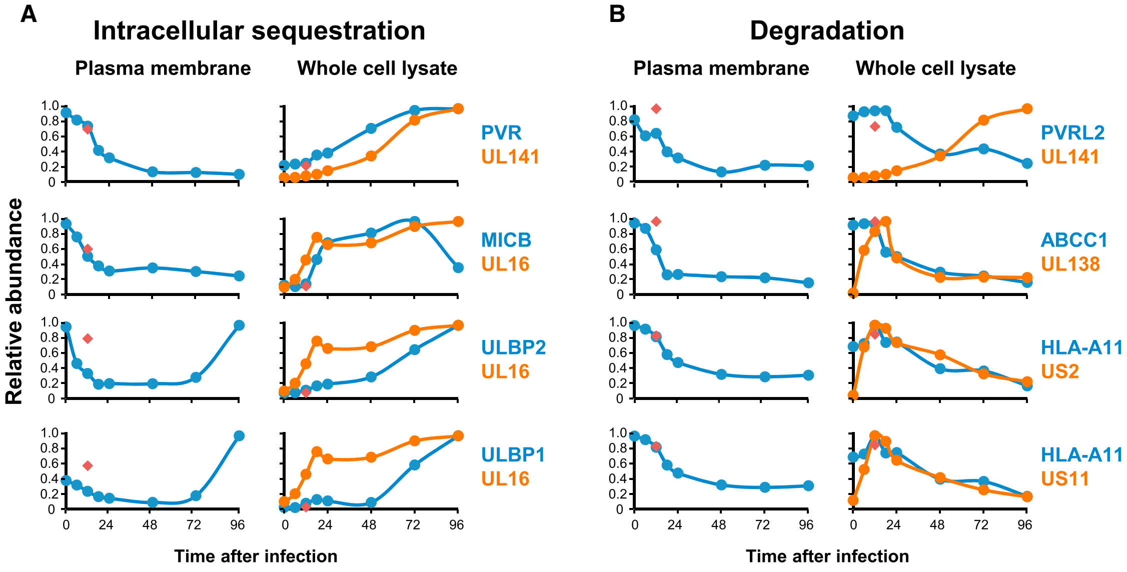

Figure 7. QTV Provides Mechanistic Insights into Downregulated Cell-Surface Targets

(A) Proteins that are known to be sequestered within the cell accumulated in WCL samples during infection.

(B) Proteins targeted for lysosomal or proteasomal degradation declined during infection.

Red diamonds, 12 hr after infection with irradiated HCMV. See also Figures S7A and S7B.

responsible for target modulation and in some cases indicating mechanism. The utility of QTV could be extended by simultaneous transcriptomic studies; if a host protein is downregulated, is this due to diminished gene expression, or posttranscriptional effects such as protein degradation?

In their recent ribosomal profiling (RP) analysis of HCMV, Stern-Ginossar et al. (2012) identified 147/171 canonical genes. We quantified 139/171 proteins (Table S2), notably including 15 genes not detected by RP. Proteins UL1 and UL6 were quantified at the PM but not in WCL samples suggesting that overall abundance of both mRNA and protein might be low. It is unclear why RP failed to detect the 13 remaining proteins, which in several cases we quantified by abundant peptides. It is likely that the sensitivity of QTV for low-abundant proteins will be further increased with the next generation of Orbitrap mass spectrometers, enabling an even more comprehensive coverage of both HCMV and human proteomes. For example, analysis of experiment WCL3 using the new Orbitrap Fusion enabled quantitation of five noncanonical ORFs we previously did not detect. Even with this sensitive instrument, it is still possible that we were unable to detect very low-abundant proteins, which may explain why some studies have detected viral transcripts earlier in infection that our protein measurements suggest. An increase in proteome coverage will also improve our ability to distinguish protein isoforms, which complicated our quantitation of UL122.

With an increasing frequency of transplantation and emergence of drug resistance, novel strategies are required to treat HCMV infection. QTV provides evidence for up to 29 cell-surface viral glycoproteins that can now be assessed as targets for therapeutic monoclonal or bispecific antibodies, to stimulate antibody-dependent cellular cytotoxicity or deliver a cytotoxin. Strongly upregulated PM proteins may constitute alternative antibody targets. By directing treatment toward proteins expressed early in infection, it may be possible to eliminate infected cells prior to the release of infectious virus. The efficacy and safety of such an approach is suggested by small-scale treatment of patients with severe drug-resistant HCMV disease using pooled immunoglobulin selected for high antibody titers to HCMV (Alexander et al., 2010). QTV has demonstrated great utility in being able to identify and quantify the regulation of virus and host proteins on the cell surface without requirement for specific antibodies. Clearly, the cell-surface viral proteins we describe require validation using an independent approach once reagents are available.

QTV is applicable to any virus, or indeed any intracellular pathogen with a robust in vitro model and has the potential to improve our understanding of infection.

EXPERIMENTAL PROCEDURES

Brief descriptions of key experimental procedures are provided below. For complete details, see Extended Experimental Procedures.

Virus Infections

Twenty-four hours prior to each infection, 1.5×10^7 HFFFs were plated in a 150 cm^2 flask. Cells were sequentially infected at multiplicity of infection 10 with HCMV strain Merlin. Greater than 95% of cells were routinely infected using this approach (Figures S4A and S4B). Infections were staggered such that all flasks were harvested simultaneously. For 12 hr irradiated virus infection, virus was gamma irradiated with a dose of 3,500 Gy.

Protein Isolation and Peptide Labeling with Tandem Mass Tags

Plasma membrane profiling was performed as described previously (Weekes et al., 2012). One hundred percent of each peptide sample was labeled with TMT reagent, and six fractions were generated from combined peptide samples by tip-based strong cation exchange. For whole-proteome analysis, cells

were lysed and protein was reduced and then alkylated. Protein was digested with LysC (experiment 1) or LysC and then Trypsin (experiments 2 and 3). Peptides were labeled with TMT reagent, and 12 fractions were generated by high-pH reverse-phase HPLC.

Mass Spectrometry and Data Analysis

We performed mass spectrometry using an Orbitrap Elite (experiments 1 and 2) or an Orbitrap Fusion (experiment WCL3) and quantified TMT reporter ions from the MS3 scan (McAlister et al., 2012; Ting et al., 2011). Peptides were identified and quantified using a Sequest-based in-house software pipeline. A combined database was searched, consisting of (1) human Uniprot, (2) Merlin strain HCMV Uniprot, and (3) all additional noncanonical HCMV ORFs (Stern-Ginossar et al., 2012). Peptide-spectral matches were filtered to a 1% false discovery rate (FDR) using linear discriminant analysis in conjunction with the target-decoy method (Huttlin et al., 2010). The resulting data set was further collapsed to a final protein-level FDR of 1%. Protein assembly was guided by principles of parsimony. Where all PSM from a given HCMV protein could be explained either by a canonical gene or noncanonical ORF, the canonical gene was picked in preference. Proteins were quantified by summing TMT reporter ion counts across all matching PSM after filtering based on isolation specificity (Pease et al., 2013). Reverse and contaminant proteins were removed, and protein quantitation values were exported for normalization and further analysis in Excel. Hierarchical clustering was performed using centroid linkage with Pearson correlation unless otherwise noted. One-way ANOVA was used to identify proteins differentially expressed over time in experiments PM1 and WCL1, and p values were corrected for multiple testing using the method of Benjamini-Hochberg. Other statistical analysis was performed using XLStat.

NK and T Cell CD107a Mobilization Assays

NK and T cell degranulation assays were performed in a similar manner to that described previously (Prod'homme et al., 2010). These protocols were approved by the Cardiff University School of Medicine Ethics Committee Ref. no: 10/20.

SUPPLEMENTAL INFORMATION

Supplemental Information includes Extended Experimental Procedures, seven figures, one data file, and seven tables and can be found with this article online at http://dx.doi.org/10.1016/j.cell.2014.04.028.

AUTHOR CONTRIBUTIONS

M.P.W. and P.T. designed the experiments and wrote the manuscript. M.P.W., P.T., C.A.F., R.J.S., E.C.Y.W., R.A., and I.M. performed the experiments. M.P.W., E.L.H, and D.N. analyzed the proteomics data. E.L.H., C.A.F., D.N., R.J.S., E.C.Y.W., G.W.G.W., P.J.L., and S.P.G. edited the manuscript. G.W.G.W., P.J.L., and S.P.G. supervised all research.

ACKNOWLEDGMENTS

We are grateful to Dr. Andrew Davison for assistance with the assembly of HCMV FASTA files; to Joao Paulo, Ryan Kunz, and Martin Wuehr for mass spectrometry and to Alexander Devaux for technical assistance. This work was supported by a Wellcome Trust Fellowship (093966/Z/10/Z) to M.P.W.; an MRC Project Grant and a Wellcome Trust Programme Grant (G1000236, WT090323MA) to G.W.G.W., P.T., and E.C.Y.W.; a Wellcome Trust Senior Fellowship (084957/Z/08/Z) to P.J.L. and National Institute of Health grant (GM067945) to S.P.G. This study was additionally supported by the Cambridge Biomedical Research Centre, UK. We have filed a provisional patent based on our findings.

Received: November 4, 2013
Revised: February 18, 2014
Accepted: April 3, 2014
Published: June 5, 2014

REFERENCES

Alexander, B.T., Hladnik, L.M., Augustin, K.M., Casabar, E., McKinnon, P.S., Reichley, R.M., Ritchie, D.J., Westervelt, P., and Dubberke, E.R. (2010). Use of cytomegalovirus intravenous immune globulin for the adjunctive treatment of cytomegalovirus in hematopoietic stem cell transplant recipients. Pharmacotherapy *30*, 554–561.

Alwine, J.C. (2012). The human cytomegalovirus assembly compartment: a masterpiece of viral manipulation of cellular processes that facilitates assembly and egress. PLoS Pathog. *8*, e1002878.

Amsler, L., Verweij, M.C., and DeFilippis, V.R. (2013). The tiers and dimensions of evasion of the type I interferon response by human cytomegalovirus. J. Mol. Biol. *425*, 4857–4871.

Angelova, M., Zwezdaryk, K., Ferris, M., Shan, B., Morris, C.A., and Sullivan, D.E. (2012). Human cytomegalovirus infection dysregulates the canonical Wnt/β-catenin signaling pathway. PLoS Pathog. *8*, e1002959.

Bonneville, M., O'Brien, R.L., and Born, W.K. (2010). Gammadelta T cell effector functions: a blend of innate programming and acquired plasticity. Nat. Rev. Immunol. *10*, 467–478.

Ceeraz, S., Nowak, E.C., and Noelle, R.J. (2013). B7 family checkpoint regulators in immune regulation and disease. Trends Immunol. *34*, 556–563.

Chambers, J., Angulo, A., Amaratunga, D., Guo, H., Jiang, Y., Wan, J.S., Bittner, A., Frueh, K., Jackson, M.R., Peterson, P.A., et al. (1999). DNA microarrays of the complex human cytomegalovirus genome: profiling kinetic class with drug sensitivity of viral gene expression. J. Virol. *73*, 5757–5766.

Duggal, N.K., and Emerman, M. (2012). Evolutionary conflicts between viruses and restriction factors shape immunity. Nat. Rev. Immunol. *12*, 687–695.

Dunn, C., Chalupny, N.J., Sutherland, C.L., Dosch, S., Sivakumar, P.V., Johnson, D.C., and Cosman, D. (2003a). Human cytomegalovirus glycoprotein UL16 causes intracellular sequestration of NKG2D ligands, protecting against natural killer cell cytotoxicity. J. Exp. Med. *197*, 1427–1439.

Feng, X., Schröer, J., Yu, D., and Shenk, T. (2006). Human cytomegalovirus pUS24 is a virion protein that functions very early in the replication cycle. J. Virol. *80*, 8371–8378.

Gariano, G.R., Dell'Oste, V., Bronzini, M., Gatti, D., Luganini, A., De Andrea, M., Gribaudo, G., Gariglio, M., and Landolfo, S. (2012). The intracellular DNA sensor IFI16 gene acts as restriction factor for human cytomegalovirus replication. PLoS Pathog. *8*, e1002498.

Hansen, S.G., Piatak, M., Jr., Ventura, A.B., Hughes, C.M., Gilbride, R.M., Ford, J.C., Oswald, K., Shoemaker, R., Li, Y., Lewis, M.S., et al. (2013). Immune clearance of highly pathogenic SIV infection. Nature *502*, 100–104.

Huang, W., Sherman, B.T., and Lempicki, R.A. (2009). Systematic and integrative analysis of large gene lists using DAVID bioinformatics resources. Nat. Protoc. *4*, 44–57.

Hubo, M., Trinschek, B., Kryczanowsky, F., Tuettenberg, A., Steinbrink, K., and Jonuleit, H. (2013). Costimulatory molecules on immunogenic versus tolerogenic human dendritic cells. Front Immunol *4*, 82.

Hunter, S., Jones, P., Mitchell, A., Apweiler, R., Attwood, T.K., Bateman, A., Bernard, T., Binns, D., Bork, P., Burge, S., et al. (2012). InterPro in 2011: new developments in the family and domain prediction database. Nucleic Acids Res. *40* (Database issue), D306–D312.

Huttlin, E.L., Jedrychowski, M.P., Elias, J.E., Goswami, T., Rad, R., Beausoleil, S.A., Villén, J., Haas, W., Sowa, M.E., and Gygi, S.P. (2010). A tissue-specific atlas of mouse protein phosphorylation and expression. Cell *143*, 1174–1189.

Khan, Z., Yaiw, K.C., Wilhelmi, V., Lam, H., Rahbar, A., Stragliotto, G., and Söderberg-Nauclér, C. (2014). Human cytomegalovirus immediate early proteins promote degradation of connexin 43 and disrupt gap junction communication: implications for a role in gliomagenesis. Carcinogenesis *35*, 145–154.

Kim, Y.E., Lee, J.H., Kim, E.T., Shin, H.J., Gu, S.Y., Seol, H.S., Ling, P.D., Lee, C.H., and Ahn, J.H. (2011a). Human cytomegalovirus infection causes degradation of Sp100 proteins that suppress viral gene expression. J. Virol. *85*, 11928–11937.

Koyuncu, E., Purdy, J.G., Rabinowitz, J.D., and Shenk, T. (2013). Saturated very long chain fatty acids are required for the production of infectious human cytomegalovirus progeny. PLoS Pathog. *9*, e1003333.

Le, V.T., Trilling, M., Wilborn, M., Hengel, H., and Zimmermann, A. (2008). Human cytomegalovirus interferes with signal transducer and activator of transcription (STAT) 2 protein stability and tyrosine phosphorylation. J. Gen. Virol. *89*, 2416–2426.

Ma, Y., Wang, N., Li, M., Gao, S., Wang, L., Zheng, B., Qi, Y., and Ruan, Q. (2012). Human CMV transcripts: an overview. Future Microbiol. *7*, 577–593.

McAlister, G.C., Huttlin, E.L., Haas, W., Ting, L., Jedrychowski, M.P., Rogers, J.C., Kuhn, K., Pike, I., Grothe, R.A., Blethrow, J.D., and Gygi, S.P. (2012). Increasing the multiplexing capacity of TMTs using reporter ion isotopologues with isobaric masses. Anal. Chem. *84*, 7469–7478.

Mocarski E.S., Shenk T., Griffiths P.D., and Pass R.F., eds. (2013). Cytomegaloviruses, 6th Edition (Philadelphia: Lipincott Williams and Wilkins).

Nichols, W.G., Corey, L., Gooley, T., Davis, C., and Boeckh, M. (2002). High risk of death due to bacterial and fungal infection among cytomegalovirus (CMV)-seronegative recipients of stem cell transplants from seropositive donors: evidence for indirect effects of primary CMV infection. J. Infect. Dis. *185*, 273–282.

Nikolov, D.B., Xu, K., and Himanen, J.P. (2013). Eph/ephrin recognition and the role of Eph/ephrin clusters in signaling initiation. Biochim. Biophys. Acta *1834*, 2160–2165.

Omoto, S., and Mocarski, E.S. (2014). Transcription of true late (γ2) cytomegalovirus genes requires UL92 function that is conserved among beta- and gammaherpesviruses. J. Virol. *88*, 120–130.

Paludan, S.R., Bowie, A.G., Horan, K.A., and Fitzgerald, K.A. (2011). Recognition of herpesviruses by the innate immune system. Nat. Rev. Immunol. *11*, 143–154.

Pease, B.N., Huttlin, E.L., Jedrychowski, M.P., Talevich, E., Harmon, J., Dillman, T., Kannan, N., Doerig, C., Chakrabarti, R., Gygi, S.P., and Chakrabarti, D. (2013). Global analysis of protein expression and phosphorylation of three stages of Plasmodium falciparum intraerythrocytic development. J. Proteome Res. *12*, 4028–4045.

Powers, C., DeFilippis, V., Malouli, D., and Früh, K. (2008). Cytomegalovirus immune evasion. Curr. Top. Microbiol. Immunol. *325*, 333–359.

Prod'homme, V., Sugrue, D.M., Stanton, R.J., Nomoto, A., Davies, J., Rickards, C.R., Cochrane, D., Moore, M., Wilkinson, G.W., and Tomasec, P. (2010). Human cytomegalovirus UL141 promotes efficient downregulation of the natural killer cell activating ligand CD112. J. Gen. Virol. *91*, 2034–2039.

Rahm, N., and Telenti, A. (2012). The role of tripartite motif family members in mediating susceptibility to HIV-1 infection. Curr Opin HIV AIDS *7*, 180–186.

Schoggins, J.W., Wilson, S.J., Panis, M., Murphy, M.Y., Jones, C.T., Bieniasz, P., and Rice, C.M. (2011). A diverse range of gene products are effectors of the type I interferon antiviral response. Nature *472*, 481–485.

Scott, I. (2009). Degradation of RIG-I following cytomegalovirus infection is independent of apoptosis. Microbes Infect. *11*, 973–979.

Stanton, R.J., McSharry, B.P., Rickards, C.R., Wang, E.C., Tomasec, P., and Wilkinson, G.W. (2007). Cytomegalovirus destruction of focal adhesions revealed in a high-throughput Western blot analysis of cellular protein expression. J. Virol. *81*, 7860–7872.

Stenberg, R.M., Depto, A.S., Fortney, J., and Nelson, J.A. (1989). Regulated expression of early and late RNAs and proteins from the human cytomegalovirus immediate-early gene region. J. Virol. *63*, 2699–2708.

Stern-Ginossar, N., Weisburd, B., Michalski, A., Le, V.T., Hein, M.Y., Huang, S.X., Ma, M., Shen, B., Qian, S.B., Hengel, H., et al. (2012). Decoding human cytomegalovirus. Science *338*, 1088–1093.

Subramanian, A., Tamayo, P., Mootha, V.K., Mukherjee, S., Ebert, B.L., Gillette, M.A., Paulovich, A., Pomeroy, S.L., Golub, T.R., Lander, E.S., and Mesirov, J.P. (2005). Gene set enrichment analysis: a knowledge-based approach for interpreting genome-wide expression profiles. Proc. Natl. Acad. Sci. USA *102*, 15545–15550.

Sullivan, R., Fassolitis, A.C., Larkin, E.P., Read, R.B., Jr., and Peeler, J.T. (1971). Inactivation of thirty viruses by gamma radiation. Appl. Microbiol. *22*, 61–65.

Takamatsu, H., and Kumanogoh, A. (2012). Diverse roles for semaphorin-plexin signaling in the immune system. Trends Immunol. *33*, 127–135.

Terhune, S.S., Moorman, N.J., Cristea, I.M., Savaryn, J.P., Cuevas-Bennett, C., Rout, M.P., Chait, B.T., and Shenk, T. (2010). Human cytomegalovirus UL29/28 protein interacts with components of the NuRD complex which promote accumulation of immediate-early RNA. PLoS Pathog. *6*, e1000965.

Ting, L., Rad, R., Gygi, S.P., and Haas, W. (2011). MS3 eliminates ratio distortion in isobaric multiplexed quantitative proteomics. Nat. Methods *8*, 937–940.

Tomasec, P., Wang, E.C., Davison, A.J., Vojtesek, B., Armstrong, M., Griffin, C., McSharry, B.P., Morris, R.J., Llewellyn-Lacey, S., Rickards, C., et al. (2005). Downregulation of natural killer cell-activating ligand CD155 by human cytomegalovirus UL141. Nat. Immunol. *6*, 181–188.

van der Wal, F.J., Kikkert, M., and Wiertz, E. (2002). The HCMV gene products US2 and US11 target MHC class I molecules for degradation in the cytosol. Curr. Top. Microbiol. Immunol. *269*, 37–55.

Varnum, S.M., Streblow, D.N., Monroe, M.E., Smith, P., Auberry, K.J., Pasa-Tolic, L., Wang, D., Camp, D.G., 2nd, Rodland, K., Wiley, S., et al. (2004). Identification of proteins in human cytomegalovirus (HCMV) particles: the HCMV proteome. J. Virol. *78*, 10960–10966.

Vavassori, S., Kumar, A., Wan, G.S., Ramanjaneyulu, G.S., Cavallari, M., El Daker, S., Beddoe, T., Theodossis, A., Williams, N.K., Gostick, E., et al. (2013). Butyrophilin 3A1 binds phosphorylated antigens and stimulates human γδ T cells. Nat. Immunol. *14*, 908–916.

Vivier, E., Tomasello, E., Baratin, M., Walzer, T., and Ugolini, S. (2008). Functions of natural killer cells. Nat. Immunol. *9*, 503–510.

Weekes, M.P., Antrobus, R., Talbot, S., Hör, S., Simecek, N., Smith, D.L., Bloor, S., Randow, F., and Lehner, P.J. (2012). Proteomic plasma membrane profiling reveals an essential role for gp96 in the cell surface expression of LDLR family members, including the LDL receptor and LRP6. J. Proteome Res. *11*, 1475–1484.

Weekes, M.P., Tan, S.Y.L., Poole, E., Talbot, S., Antrobus, R., Smith, D.L., Montag, C., Gygi, S.P., Sinclair, J.H., and Lehner, P.J. (2013). Latency-associated degradation of the MRP1 drug transporter during latent human cytomegalovirus infection. Science *340*, 199–202.

Wilkinson, G.W., Tomasec, P., Stanton, R.J., Armstrong, M., Prod'homme, V., Aicheler, R., McSharry, B.P., Rickards, C.R., Cochrane, D., Llewellyn-Lacey, S., et al. (2008). Modulation of natural killer cells by human cytomegalovirus. J. Clin. Virol. *41*, 206–212.

Witherden, D.A., Watanabe, M., Garijo, O., Rieder, S.E., Sarkisyan, G., Cronin, S.J., Verdino, P., Wilson, I.A., Kumanogoh, A., Kikutani, H., et al. (2012). The CD100 receptor interacts with its plexin B2 ligand to regulate epidermal γδ T cell function. Immunity *37*, 314–325.

Supplemental Information

EXTENDED EXPERIMENTAL PROCEDURES

Materials
Tandem mass tag (TMT) 8-plex and 10-plex isobaric reagents were from Thermo Scientific, (Rockford, IL) (Viner et al., 2013). Water and organic solvents were form J.T. Baker (Center Valley, PA). Unless otherwise noted, all other chemicals were from Sigma-Aldrich (St. Louis, MO).

Cells and Viruses
Primary human fetal foreskin fibroblast cells (HFFF) were grown in Dulbecco's modified eagles medium (DMEM) (Life Technologies) supplemented with fetal bovine serum (10% v/v), penicillin/streptomycin and L-glutamine (GIBCO) at 37°C in 5% CO_2. Cells were verified to be mycoplasma negative. The HCMV strain Merlin is designated the reference HCMV genome sequence by the National Center for Biotechnology Information and was sequenced after only 3 passages in vitro (Dolan et al., 2004). We recently constructed a BAC clone containing the complete Merlin genome to provide a reproducible source of genetically intact, clonal virus for pathogenesis studies (Stanton et al., 2010). Merlin BAC derived clone RCMV1111 used for this study contains point mutations in RL13 and UL128, enhancing replication in fibroblasts (Stanton et al., 2010). Generation of virus stocks by BAC transfection into fibroblasts was as previously described (Stanton et al., 2010). Virus was gamma irradiated with a dose of 3500Gy using a Gammacell 1000 Elite (Nordion International), and inactivation was verified by absence of immunofluorescence for IE1 compared to control (data not shown).

Virus Infections
24h prior to each infection, 1.5×10^7 HFFFs were plated in a 150cm^2 flask. Cells were sequentially infected at multiplicity of infection 10 with HCMV strain Merlin. Infections were staggered such that all flasks were harvested simultaneously. Infection efficiency was monitored using HCMV IE1 intracellular staining or MHC class-I cell surface staining and confirmed as $\geq$ 95% (Figures S4A and S4B). For experiment WCL3, where indicated cells were incubated with Phosphonoformate (PFA) at 300ug/ml from the time of infection onward.

Plasma Membrane Profiling
Plasma membrane profiling was performed as described previously, with minor modifications for adherent cells (Weekes et al., 2010; Weekes et al., 2012). Briefly, one 150cm2 flask of HCMV-infected HFFFs per condition was washed twice with ice-cold PBS. Sialic acid residues were oxidized with sodium meta-periodate (Thermo) then biotinylated with aminooxy-biotin (Biotium). The reaction was quenched, and the biotinylated cells scraped into 1% Triton X-100 lysis buffer. Biotinylated glycoproteins were enriched with high-affinity streptavidin agarose beads (Pierce) and washed extensively. Captured protein was denatured with DTT, alkylated with iodoacetamide (IAA, Sigma) and digested on-bead with trypsin (Promega) in 100 mM HEPES pH 8.5 for 3h. Tryptic peptides were collected. Ting et al. (2011) suggest that LysC digestion is preferable for quantitative proteomic analysis using TMT, because it guarantees labeling of both N-termini and C-terminal lysine side chains on virtually every peptide (Ting et al., 2011). In practice TMT labeling is compatible with analysis of even tryptic peptides that may only bear TMT labels on their N terminus.

Whole-Proteome Samples
Cells were washed twice with PBS, and 1 ml lysis buffer added (experiment 1: 8 M Urea/100 mM HEPES pH8.5, experiments 2, 3: 6 M Guanidine/50 mM HEPES pH8.5). Cell lifters (Corning) were used to scrape cells in lysis buffer, which was removed to an eppendorf tube, vortexed extensively then sonicated. Cell debris was removed by centrifuging at 13,000 g for 10 min twice. Protein concentrations were determined by BCA assay (Pierce). Dithiothreitol (DTT) was added to a final concentration of 5 mM and samples were incubated for 20 min. Cysteines were alkylated with 15 mM iodoacetamide and incubated 20 min at room temperature in the dark. Excess iodoacetamide was quenched with DTT for 15 min. Samples were diluted with 100 mM HEPES pH 8.5 to 4 M Urea (experiment 1) or 1.5 M Guanidine (experiments 2, 3) followed by digestion at room temperature for 3 hr with LysC protease at a 1:100 protease-to-protein ratio. For experiments 2 and 3, trypsin was then added at a 1:100 protease-to-protein ratio followed by overnight incubation at 37°C. The reaction was quenched with 1% formic acid, subjected to C18 solid-phase extraction (Sep-Pak, Waters) and vacuum-centrifuged to near-dryness. While experiment 1 employed LysC digestion as recommended by Ting et al. (2011), experiments 2 and 3 employed a modified protocol for two reasons: (i) Guanidine was found to solubilize membrane proteins better than Urea, which would facilitate comparisons with the PM data; and (ii) sequential digestion with LysC followed by Trypsin was found to provide more robust digestion across samples, frequently resulting in improved depth of proteome coverage.

Peptide Labeling with Tandem Mass Tags
In preparation for TMT labeling, desalted peptides were dissolved in 100 mM HEPES pH 8.5. For whole proteome samples, peptide concentration was measured by microBCA (Pierce), and 100 μg of peptide labeled with TMT reagent. For plasma membrane samples, 100% of each peptide sample was labeled.

TMT reagents (0.8 mg) were dissolved in 40 μl anhydrous acetonitrile and 10 μl (whole proteome) or 2.5 μl (PM samples) added to peptide at a final acetonitrile concentration of 30% (v/v). For experiments PM1 and WCL1, samples were labeled as follows: mock

replicate 1 (TMT 126); mock replicate 2 (TMT 128); 24h infection replicate 1 (TMT 127n); 24h infection replicate 2 (TMT 127c); 48h infection replicate 1 (TMT 129n); 48h infection replicate 2 (TMT 129c); 72h infection replicate 1 (TMT 130); 72h infection replicate 2 (TMT 131). For experiments PM2 and WCL2, samples were labeled as described in Figure S1D. For experiment WCL3, samples were labeled as follows: mock (TMT 126); mock + PFA (TMT 131); 24h infection (TMT 127n); 24h infection + PFA (TMT 127c); 48h infection (TMT 128n); 48h infection + PFA (TMT 128c); 72h infection (TMT 129n); 72h infection + PFA (TMT 129c); 96h infection (TMT 130n); 96h infection + PFA (TMT 130c). Following incubation at room temperature for 1 hr, the reaction was quenched with hydroxylamine to a final concentration of 0.3% (v/v). TMT-labeled samples were combined at a 1:1:1:1:1:1:1:1 ratio (8-plex TMT, experiment 1) or 1:1:1:1:1:1:1:1:1:1 ratio (10-plex TMT, experiments 2, 3). The sample was vacuum-centrifuged to near dryness and subjected to C18 solid-phase extraction (SPE) (Sep-Pak, Waters).

Offline High pH Reversed-Phase Fractionation, Whole-Proteome Samples

Combined, TMT-labeled peptide samples were fractionated using an Agilent 300Extend C18 column (5 μm particles, 4.6 mm ID, 220 mm length) and an Agilent 1100 quaternary pump equipped with a degasser and a photodiode array detector (220 and 280nm, ThermoFisher, Waltham, MA). Peptides were separated with a gradient of 5% to 35% acetonitrile in 10 mM ammonium bicarbonate pH 8 over 60 min. 96 resulting fractions were consolidated into 12, acidified to 1% formic acid and vacuum-centrifuged to near dryness. Each fraction was desalted using a StageTip (Rappsilber et al., 2007), dried, and reconstituted in 4% acetonitrile / 5% formic acid prior to LC-MS/MS.

Offline Tip-Based Strong Cation Exchange SCX Fractionation, PM Samples

Our previously described protocol for solid-phase extraction based SCX peptide fractionation (Dephoure and Gygi, 2011) was modified for small peptide amounts. Briefly, 10 mg of PolySulfethyl A bulk material (Nest Group Inc) was loaded on to a fritted 200ul tip in 100% Methanol using a vacuum manifold. SCX material was conditioned slowly with 1 ml SCX buffer A (7 mM KH_2PO_4, pH 2.65, 30% Acetonitrile), then 0.5 ml SCX buffer B (7 mM KH_2PO_4, pH 2.65, 350 mM KCl, 30% Acetonitrile) then 2 ml SCX buffer A. Dried peptides were resuspended in 500 μl SCX buffer A and added to the tip at a flow rate of ~150 μl/min, followed by a 150 μl wash with SCX buffer A. Fractions were eluted in 150ul buffer at increasing K^+ concentrations (10, 25, 40, 60, 90, 150 mM KCl), vacuum-centrifuged to near dryness then desalted using StageTips.

Liquid Chromatography and Tandem Mass Spectrometry

Mass spectrometry data were acquired using an Orbitrap Elite mass spectrometer (experiments PM1, PM2, WCL1, WCL2) coupled with a Proxeon EASY-nLC II liquid chromatography (LC) pump or an Orbitrap Fusion (experiment WCL3) coupled with a Proxeon EASY-nLC 1000 LC pump (Thermo Fisher Scientific, San Jose, CA).

For Orbitrap Elite Experiments

peptides were separated on a 100 μm inner diameter microcapillary column packed with 0.5 cm of Magic C4 resin (5 μm, 100 Å, Michrom Bioresources) followed by approximately 20 cm of Maccel C18 resin (3 μm, 200 Å, Nest Group).

Peptides were separated using a 3 hr gradient (WCL samples) or 2h gradient (PM samples) of 6 to 30% acetonitrile in 0.125% formic acid at a flow rate of 300 nl/min. Each analysis used an MS3-based TMT method (McAlister et al., 2012; Ting et al., 2011). The scan sequence began with an MS1 spectrum (Orbitrap analysis, resolution 60,000, 300−1500 Th, AGC target 1×10^6, maximum injection time 150 ms). The top ten precursors were then selected for MS2/MS3 analysis. MS2 analysis consisted of CID (quadrupole ion trap analysis, AGC 2×10^3, NCE 35, q-value 0.25, maximum injection time 100 ms). MS3 precursors were fragmented by HCD prior to Orbitrap analysis (NCE 50, max AGC 1.5×10^5, maximum injection time 250 ms, isolation specificity 2.5 Th, resolution 30,000) (McAlister et al., 2012).

For Orbitrap Fusion Experiment WCL3

peptides were separated on a 75 μm inner diameter microcapillary column packed with 0.5 cm of Magic C4 resin (5 μm, 100 Å, Michrom Bioresources) followed by approximately 20 cm of GP118 resin (1.8 μm, 120 Å, Sepax Technologies).

Peptides were separated using a 3 hr gradient (WCL samples) or 2h gradient (PM samples) of 6 to 30% acetonitrile in 0.125% formic acid at a flow rate of 300 nl/min. Each analysis used an MS3-based TMT method (McAlister et al., 2012; Ting et al., 2011). The scan sequence began with an MS1 spectrum (Orbitrap analysis, resolution 120,000, 400−1400 Th, AGC target 2×10^5, maximum injection time 200 ms). 'Top speed' (2 s) was selected for MS2 analysis, which consisted of CID (quadrupole ion trap analysis, AGC 4×10^3, NCE 35, maximum injection time 150 ms). The top ten precursors were selected for MS3 analysis, in which precursors were fragmented by HCD prior to Orbitrap analysis (NCE 55, max AGC 5×10^4, maximum injection time 250 ms, isolation specificity 0.5 Th, resolution 60,000) (McAlister et al., 2012).

Data Analysis

Mass spectra were processed using a Sequest-based in-house software pipeline. MS spectra were converted to mzXML using a modified version of ReAdW.exe. A combined database was constructed from (a) the human Uniprot database (August 10, 2011), (b) the human cytomegalovirus (strain Merlin) Uniprot database, (c) all additional novel human cytomegalovirus ORFs described by Stern-Ginossar et al. (2012) and (d) common contaminants such as porcine trypsin and endoproteinase LysC. The combined database was concatenated with a reverse database composed of all protein sequences in reversed order. Searches were performed using a 20 ppm precursor ion tolerance. Product ion tolerance was set to 0.03 Th. TMT tags on lysine residues and peptide N termini

(229.162932 Da) and carbamidomethylation of cysteine residues (57.02146 Da) were set as static modifications, while oxidation of methionine residues (15.99492 Da) was set as a variable modification.

To control the fraction of erroneous protein identifications, we used a target-decoy strategy (Elias and Gygi, 2007, 2010). Peptide spectral matches (PSMs) were filtered to an initial peptide-level false discovery rate (FDR) of 1% with subsequent filtering to attain a final protein-level FDR of 1%. PSM filtering was performed using a linear discriminant analysis, as described previously (Huttlin et al., 2010), considering the following parameters: XCorr, ΔCn, missed cleavages, peptide length, charge state, and precursor mass accuracy. Protein assembly was guided by principles of parsimony to produce the smallest set of proteins necessary to account for all observed peptides. Where all PSMs from a given HCMV protein could be explained either by a canonical gene or novel ORF, the canonical gene was picked in preference.

Proteins were quantified by summing TMT reporter ion counts across all matching peptide-spectral matches using in-house software, as described previously (Pease et al., 2013). Briefly, a 0.003 Th window around the theoretical m/z of each reporter ion (126, 127n, 127c, 128n, 128c, 129n, 129c, 130n, 130c, 131) was scanned for ions, and the maximum intensity nearest to the theoretical m/z was used. The primary determinant of quantitation quality is the number of TMT reporter ions detected in each MS3 spectrum, which is directly proportional to the signal-to-noise ratio observed for each ion (Makarov and Denisov, 2009). Conservatively, we require every individual peptide used for quantitation to contribute sufficient TMT reporter ions (minimum of ~500 per spectrum) so that each on its own is expected to provide a representative picture of relative protein abundance (McAlister et al., 2012). We additionally employ an isolation specificity filter to minimize peptide coisolation (Ting et al., 2011). Peptide-spectral matches with poor quality MS3 spectra (more than 9 TMT channels missing and/or a combined signal:noise ratio of less than 100 across all TMT reporter ions) or no MS3 spectra at all were excluded from quantitation. All MS2 and MS3 spectra from novel ORFs were all manually validated to confirm both identifications and quantifications. Protein quantitation values were exported for further analysis in Excel.

For protein quantitation, reverse and contaminant proteins were removed, then each reporter ion channel was summed across all quantified proteins and normalized assuming equal protein loading across all 8 or 10 samples. Gene Ontology and KEGG terms (Ashburner et al., 2000; Kanehisa and Goto, 2000) were added using Perseus version 1.4.1.3 (Cox et al., 2009). Gene name aliases were added using GeneALaCart (www.genecards.org) (Rebhan et al., 1997). The one-way ANOVA test was used to identify proteins differentially expressed over time in experiments PM1 and WCL1, and was corrected using the method of Benjamini-Hochberg to control for multiple testing error (Benjamini and Hochberg, 1995). A Benjamini-Hochberg-corrected p value < 0.05 was considered statistically significant. Values were calculated using Mathematica (Wolfram Research). Other statistical analyses including Principal Component analysis and k-means clustering were performed using XLStat (Addinsoft). Hierarchical centroid clustering based on uncentered Pearson correlation was performed using Cluster 3.0 (Stanford University) and visualized using Java Treeview (http://jtreeview.sourceforge.net) unless otherwise noted. For RNaseq data from Stern-Ginosar et al. (2007), mRNA reads densities from 5, 24 and 72h for each transcript were normalized to 1, and hierarchical clustering based on Euclidian distance was performed using Cluster 3.0. For comparison of experiment WCL1 to Powerblot data from Stanton et al. (2007), we compared fold change at 72h for the proteins quantified by Powerblot at the highest confidence (levels 7, 8, 9, 10 [Stanton et al., 2007]) and determined association between protein changes using Fisher's exact test.

Pathway Analysis

The Database for Annotation, Visualization and Integrated Discovery (DAVID) was used to determine protein family and KEGG pathway enrichment (Huang et al., 2009). A given cluster was always searched against a background of all proteins quantified within the relevant experiment. To generate KEGG pathway diagrams, human pathway information was downloaded from the KEGG database and was imported into Mathematica 9.0 (Wolfram Research), where pathways were plotted and colored according to the designation in Figures S3A and S3B.

Gene Set Enrichment Analysis

Gene Set Enrichment Analysis (Mootha et al., 2003; Subramanian et al., 2005) was performed using the javaGSEA application provided by the Broad Institute (https://www.broadinstitute.org/gsea/index.jsp). A gene set composed of the KEGG and Reactome (Croft, 2013) gene sets provided in MSigDB (c2.cp.kegg.v4.0.symbols.gmt and c2.cp.reactome.v4.0.symbols.gmt) was searched. To generate prototypical expression profiles, the mean expression values for the k-means groupings using k = 3 was calculated for both whole-cell lysate and plasma membrane protein expression values. For each of these prototype profiles, GSEA was run using Pearson correlation to the prototype as a test statistic. Because there was only one sample per time point, p-values were generated by permuting the phenotype 1,000 times. The weighted enrichment statistic was used and the signal to noise metric was used to rank the genes. We selected all pathways enriched with a Bonferroni-corrected $p < 0.005$.

Flow Cytometry

HCMV Merlin-infected 6-well dishes of HFFFs were detached with TrypLE Express (Invitrogen) or HyQTase (Thermo), washed in PBS, and resuspended in PBS + 2% fetal calf serum. Cells were incubated with primary antibody for 20–30 min on ice, followed by anti-mouse AF647 (Molecular Probes), and fixed with 1% paraformaldehyde. The following antibodies were used: anti-PD-L1 (clone 29E.2A3, Biolegend), anti-PD-L2 (clone MIH18, Biolegend), anti-CD276 (clone DCN.70, Biolegend), anti-CEACAM1 (clone ASL-32, Biolegend), anti-CD99 (clone HCD99, Biolegend), anti-Cadherin 11 (clone 667034, R and D systems), anti-MHC class I (clone

W6-32, Serotec), anti-PCDHGC3 (cat no. ab89520, Abcam), anti-FAT1 (cat no. HPA023882, Sigma) and anti-ROR1 (clone 2H6, Abcam). Samples were acquired with a FACS Accuri flow cytometer and analyzed with FlowJo software (Tree Star).

For intracellular flow cytometry of HCMV IE1, infected cells were trypsinised 24h after infection, fixed in 4% paraformaldehyde then permeabilised using Triton X-100. Fixed cells were incubated with BSA/human serum for 30 min, followed by anti-iE1 antibody (Thermo MA1-7596) for 30 min then anti-mouse AF647.

Immunoblotting

Guanidine whole-cell lysates were precipitated using a ProteoExtract protein precipitation kit (Calbiochem). Proteins were re-dissolved in 2% SDS/Tris 200 mM pH8.5 with sonication. Protein concentration was measured by BCA (Pierce) then lysates were reduced with 100 mM DTT in loading buffer for 20 min at 65°C. Equal protein amounts were separated by SDS/PAGE using 4%–12% Bis-Tris polyacrylamide gels (Life Technologies), then transferred to PVDF membranes using the iBlot transfer system (Life Technologies). The following primary antibodies were used: anti-IFIT1 (clone 3G8, Lifespan), anti-IFIT3 (cat no. GTX112442, GeneTex), anti-MX1 (clone 2G12, GeneTex), anti-Calnexin (cat no. C142768, Lifespan), anti-MHC class I (clone HC-10, Abcam), anti-CD99 (cat no. ab108297, Abcam), anti-CLEC1A (cat no. AF1704, R and D systems), anti-FAT1 (cat no. HPA023882, Sigma), anti-PCDHGC3 (cat no. sc-134416, Santa Cruz), anti-IRF3 (clone FL-425, Santa Cruz), anti-NF-kB p50/105 (cat no. sc-7178, Santa Cruz), anti-p65/RELA (cat no. sc-109, Santa Cruz), anti-Pan-Akt (clone C67E7, Cell Signaling), anti-actin (cat no. A2066, Sigma). HRP-conjugated secondary antibodies were anti-rabbit (Cell Signaling), anti-mouse (Santa Cruz) or anti-goat (Santa Cruz). Reactive bands were detected by SuperSignal West Pico substrate (Thermo).

NK Cell CD107a Mobilization Assays

NK degranulation assays were performed in a similar manner to that described previously (Prod'homme et al., 2007; Prod'homme et al., 2010). This protocol was approved by the Cardiff University School of Medicine Ethics Committee Ref. no: 10/20. Briefly, IFN-α-activated PBMC were incubated in triplicate (donors A, B) or duplicate (donor C) with target cells and anti-CD107a-FITC mAb (3 μl for 10^6 PBMC, clone H4A3, BD Biosciences) for 5h in the presence of BD GolgiStop (BD Biosciences) added 30 min after the start of the incubation. PBMC were harvested and stained with conjugated antibodies against CD3 (cat. no. 737657, Beckman Coulter) and CD56 (cat. no. A07788, Beckman Coulter), and fixed in 2% PFA before analysis by flow cytometry (BD Biosciences Accuri C Flow). siRNA-mediated knockdown of target cells was performed as instructed by the manufacturer (Life Technologies) and was confirmed by immnoblot. siRNA was obtained from QIAGEN (FAT1: cat no. SI02664424; CLEC1A: cat no. SI03100419; control: cat no. 1027280), and Lipofectamine RNAiMAX from Life Technologies (cat no. 13778-075).

Generation of HCMV-Specific T Cell Lines and T Cell CD107a Mobilization Assays

HCMV HLA-A2 restricted peptide VLEETSVML-specific T cell lines were generated by stimulating PBMC with irradiated (6000 rad) peptide-coated (100 μg/ml for 1 hr at 37°C) autologous fibroblasts in RPMI supplemented with heat-inactivated 10% fetal calf serum, 2% human AB serum (RPMI-AB) and 10 IU/ml recombinant IL-2 (rIL-2). Cultures were re-fed every 3-4 days with an equal volume of RPMI-AB containing 20 IU/ml rIL-2.

CD107 T cell mobilization assays were performed similarly to NK cell mobilization assays. Briefly, autologous fibroblasts were infected with HCMV strain Merlin at an moi of 10 for 72h then coated with VLE peptide (10 μg/ml at 37°C for 1 hr) prior to addition of effectors at an E:T ratio of 10:1. Blocking antibody experiments (anti-CEACAM1, clone ASL-32, Biolegend) were carried out in quadruplicate at 5 μg/ml, either in cultures in excess, or with pre-treatment for 10 min at 37°C before 2 washes with RPMI-10. Anti-CD107a-FITC (BD Biosciences) or isotype control was added with effectors and incubated for 1 hr before addition of BD Golgistop (BD Biosciences). Cultures were then incubated for 4 hr before cell surface staining with anti-CD8-APC (Biolegend). Samples were fixed in 2% PFA before analysis by flow cytometry (BD Biosciences Accuri C Flow).

SUPPLEMENTAL REFERENCES

Adamo, J.E., Schröer, J., and Shenk, T. (2004). Human cytomegalovirus TRS1 protein is required for efficient assembly of DNA-containing capsids. J. Virol. *78*, 10221–10229.

Ahlqvist, J., and Mocarski, E. (2011). Cytomegalovirus UL103 controls virion and dense body egress. J. Virol. *85*, 5125–5135.

Ahn, K., Angulo, A., Ghazal, P., Peterson, P.A., Yang, Y., and Früh, K. (1996). Human cytomegalovirus inhibits antigen presentation by a sequential multistep process. Proc. Natl. Acad. Sci. USA *93*, 10990–10995.

Ahn, K., Gruhler, A., Galocha, B., Jones, T.R., Wiertz, E.J., Ploegh, H.L., Peterson, P.A., Yang, Y., and Früh, K. (1997). The ER-luminal domain of the HCMV glycoprotein US6 inhibits peptide translocation by TAP. Immunity *6*, 613–621.

Anastas, J.N., and Moon, R.T. (2013). WNT signalling pathways as therapeutic targets in cancer. Nat. Rev. Cancer *13*, 11–26.

Arnon, T.I., Achdout, H., Levi, O., Markel, G., Saleh, N., Katz, G., Gazit, R., Gonen-Gross, T., Hanna, J., Nahari, E., et al. (2005). Inhibition of the NKp30 activating receptor by pp65 of human cytomegalovirus. Nat. Immunol. *6*, 515–523.

Ashburner, M., Ball, C.A., Blake, J.A., Botstein, D., Butler, H., Cherry, J.M., Davis, A.P., Dolinski, K., Dwight, S.S., Eppig, J.T., et al.; The Gene Ontology Consortium (2000). Gene ontology: tool for the unification of biology. Nat. Genet. *25*, 25–29.

Ashiru, O., Bennett, N.J., Boyle, L.H., Thomas, M., Trowsdale, J., and Wills, M.R. (2009). NKG2D ligand MICA is retained in the cis-Golgi apparatus by human cytomegalovirus protein UL142. J. Virol. *83*, 12345–12354.

Atalay, R., Zimmermann, A., Wagner, M., Borst, E., Benz, C., Messerle, M., and Hengel, H. (2002). Identification and expression of human cytomegalovirus transcription units coding for two distinct Fcgamma receptor homologs. J. Virol. *76*, 8596–8608.

Baldick, C.J., Jr., and Shenk, T. (1996). Proteins associated with purified human cytomegalovirus particles. J. Virol. *70*, 6097–6105.

Basgoz, N., Qadri, I., Navarro, D., Sears, A., Lennette, E., Youngblom, J., and Pereira, L. (1992). The amino terminus of human cytomegalovirus glycoprotein B contains epitopes that vary among strains. J. Gen. Virol. *73*, 983–988.

Benedict, C.A., Butrovich, K.D., Lurain, N.S., Corbeil, J., Rooney, I., Schneider, P., Tschopp, J., and Ware, C.F. (1999). Cutting edge: a novel viral TNF receptor superfamily member in virulent strains of human cytomegalovirus. J. Immunol. *162*, 6967–6970.

Benjamini, Y., and Hochberg, Y. (1995). Controlling the false discovery rate - a practical and powerful approach to multiple testing. J. R. Stat. Soc. Ser. B-Methodol. *57*, 289–300.

Beutler, T., Höflich, C., Stevens, P.A., Krüger, D.H., and Prösch, S. (2003). Downregulation of the epidermal growth factor receptor by human cytomegalovirus infection in human fetal lung fibroblasts. Am. J. Respir. Cell Mol. Biol. *28*, 86–94.

Bhattacharyya, S., Zagórska, A., Lew, E.D., Shrestha, B., Rothlin, C.V., Naughton, J., Diamond, M.S., Lemke, G., and Young, J.A. (2013). Enveloped viruses disable innate immune responses in dendritic cells by direct activation of TAM receptors. Cell Host Microbe *14*, 136–147.

Biegalke, B.J. (2013). Nontraditional localization and retention signals localize human cytomegalovirus pUL34 to the nucleus. J. Virol. *87*, 11939–11944.

Bodaghi, B., Jones, T.R., Zipeto, D., Vita, C., Sun, L., Laurent, L., Arenzana-Seisdedos, F., Virelizier, J.L., and Michelson, S. (1998). Chemokine sequestration by viral chemoreceptors as a novel viral escape strategy: withdrawal of chemokines from the environment of cytomegalovirus-infected cells. J. Exp. Med. *188*, 855–866.

Britt, W.J., Vugler, L., Butfiloski, E.J., and Stephens, E.B. (1990). Cell surface expression of human cytomegalovirus (HCMV) gp55-116 (gB): use of HCMV-recombinant vaccinia virus-infected cells in analysis of the human neutralizing antibody response. J. Virol. *64*, 1079–1085.

Buchkovich, N.J., Yu, Y., Pierciey, F.J., Jr., and Alwine, J.C. (2010). Human cytomegalovirus induces the endoplasmic reticulum chaperone BiP through increased transcription and activation of translation by using the BiP internal ribosome entry site. J. Virol. *84*, 11479–11486.

Casaccia-Bonnefil, P., Gu, C., and Chao, M.V. (1999). Neurotrophins in cell survival/death decisions. Adv. Exp. Med. Biol. *468*, 275–282.

Casarosa, P., Gruijthuijsen, Y.K., Michel, D., Beisser, P.S., Holl, J., Fitzsimons, C.P., Verzijl, D., Bruggeman, C.A., Mertens, T., Leurs, R., et al. (2003). Constitutive signaling of the human cytomegalovirus-encoded receptor UL33 differs from that of its rat cytomegalovirus homolog R33 by promiscuous activation of G proteins of the Gq, Gi, and Gs classes. J. Biol. Chem. *278*, 50010–50023.

Casarosa, P., Waldhoer, M., LiWang, P.J., Vischer, H.F., Kledal, T., Timmerman, H., Schwartz, T.W., Smit, M.J., and Leurs, R. (2005). CC and CX3C chemokines differentially interact with the N terminus of the human cytomegalovirus-encoded US28 receptor. J. Biol. Chem. *280*, 3275–3285.

Cassady, K.A. (2005). Human cytomegalovirus TRS1 and IRS1 gene products block the double-stranded-RNA-activated host protein shutoff response induced by herpes simplex virus type 1 infection. J. Virol. *79*, 8707–8715.

Chalupny, N.J., Rein-Weston, A., Dosch, S., and Cosman, D. (2006). Down-regulation of the NKG2D ligand MICA by the human cytomegalovirus glycoprotein UL142. Biochem. Biophys. Res. Commun. *346*, 175–181.

Chaumorcel, M., Lussignol, M., Mouna, L., Cavignac, Y., Fahie, K., Cotte-Laffitte, J., Geballe, A., Brune, W., Beau, I., Codogno, P., and Esclatine, A. (2012). The human cytomegalovirus protein TRS1 inhibits autophagy via its interaction with Beclin 1. J. Virol. *86*, 2571–2584.

Chen, Z., Knutson, E., Wang, S., Martinez, L.A., and Albrecht, T. (2007). Stabilization of p53 in human cytomegalovirus-initiated cells is associated with sequestration of HDM2 and decreased p53 ubiquitination. J. Biol. Chem. *282*, 29284–29295.

Child, S.J., Hakki, M., De Niro, K.L., and Geballe, A.P. (2004). Evasion of cellular antiviral responses by human cytomegalovirus TRS1 and IRS1. J. Virol. *78*, 197–205.

Cortese, M., Calò, S., D'Aurizio, R., Lilja, A., Pacchiani, N., and Merola, M. (2012). Recombinant human cytomegalovirus (HCMV) RL13 binds human immunoglobulin G Fc. PLoS ONE *7*, e50166.

Cosman, D., Müllberg, J., Sutherland, C.L., Chin, W., Armitage, R., Fanslow, W., Kubin, M., and Chalupny, N.J. (2001). ULBPs, novel MHC class I-related molecules, bind to CMV glycoprotein UL16 and stimulate NK cytotoxicity through the NKG2D receptor. Immunity *14*, 123–133.

Cox, J., Matic, I., Hilger, M., Nagaraj, N., Selbach, M., Olsen, J.V., and Mann, M. (2009). A practical guide to the MaxQuant computational platform for SILAC-based quantitative proteomics. Nat. Protoc. *4*, 698–705.

Cranage, M.P., Smith, G.L., Bell, S.E., Hart, H., Brown, C., Bankier, A.T., Tomlinson, P., Barrell, B.G., and Minson, T.C. (1988). Identification and expression of a human cytomegalovirus glycoprotein with homology to the Epstein-Barr virus BXLF2 product, varicella-zoster virus gpIII, and herpes simplex virus type 1 glycoprotein H. J. Virol. *62*, 1416–1422.

Croft, D. (2013). Building models using Reactome pathways as templates. Methods Mol. Biol. *1021*, 273–283.

Cui, X., Sun, Z.R., Ren, G.W., Wang, G.L., Qi, Y., Ma, Y.P., and Ruan, Q. (2011). Interaction between human cytomegalovirus UL136 protein and ATP1B1 protein. Braz. J. Med. Biol. Res. *44*, 1251–1255.

Dal Monte, P., Pignatelli, S., Mach, M., and Landini, M.P. (2001). The product of human cytomegalovirus UL73 is a new polymorphic structural glycoprotein (gpUL73). J. Hum. Virol. *4*, 26–34.

Dallman, C., Johnson, P.W., and Packham, G. (2003). Differential regulation of cell survival by CD40. Apoptosis *8*, 45–53.

Das, S., and Pellett, P.E. (2007). Members of the HCMV US12 family of predicted heptaspanning membrane proteins have unique intracellular distributions, including association with the cytoplasmic virion assembly complex. Virology *361*, 263–273.

Davison, A.J., Akter, P., Cunningham, C., Dolan, A., Addison, C., Dargan, D.J., Hassan-Walker, A.F., Emery, V.C., Griffiths, P.D., and Wilkinson, G.W. (2003). Homology between the human cytomegalovirus RL11 gene family and human adenovirus E3 genes. J. Gen. Virol. *84*, 657–663.

Dephoure, N., and Gygi, S.P. (2011). A solid phase extraction-based platform for rapid phosphoproteomic analysis. Methods *54*, 379–386.

Der, S.D., Zhou, A., Williams, B.R., and Silverman, R.H. (1998). Identification of genes differentially regulated by interferon alpha, beta, or gamma using oligonucleotide arrays. Proc. Natl. Acad. Sci. USA *95*, 15623–15628.

Dolan, A., Cunningham, C., Hector, R.D., Hassan-Walker, A.F., Lee, L., Addison, C., Dargan, D.J., McGeoch, D.J., Gatherer, D., Emery, V.C., et al. (2004). Genetic content of wild-type human cytomegalovirus. J. Gen. Virol. *85*, 1301–1312.

Dunn, W., Chou, C., Li, H., Hai, R., Patterson, D., Stolc, V., Zhu, H., and Liu, F. (2003b). Functional profiling of a human cytomegalovirus genome. Proc. Natl. Acad. Sci. USA *100*, 14223–14228.

Durfee, L.A., Lyon, N., Seo, K., and Huibregtse, J.M. (2010). The ISG15 conjugation system broadly targets newly synthesized proteins: implications for the antiviral function of ISG15. Mol. Cell *38*, 722–732.

Duttagupta, P.A., Boesteanu, A.C., and Katsikis, P.D. (2009). Costimulation signals for memory CD8+ T cells during viral infections. Crit. Rev. Immunol. *29*, 469–486.

Elias, J.E., and Gygi, S.P. (2007). Target-decoy search strategy for increased confidence in large-scale protein identifications by mass spectrometry. Nat. Methods *4*, 207–214.

Elias, J.E., and Gygi, S.P. (2010). Target-decoy search strategy for mass spectrometry-based proteomics. Methods Mol. Biol. *604*, 55–71.

Espert, L., Degols, G., Gongora, C., Blondel, D., Williams, B.R., Silverman, R.H., and Mechti, N. (2003). ISG20, a new interferon-induced RNase specific for single-stranded RNA, defines an alternative antiviral pathway against RNA genomic viruses. J. Biol. Chem. *278*, 16151–16158.

Espín, R., Roca, F.J., Candel, S., Sepulcre, M.P., González-Rosa, J.M., Alcaraz-Pérez, F., Meseguer, J., Cayuela, M.L., Mercader, N., and Mulero, V. (2013). TNF receptors regulate vascular homeostasis in zebrafish through a caspase-8, caspase-2 and P53 apoptotic program that bypasses caspase-3. Dis. Model. Mech. *6*, 383–396.

Fairley, J.A., Baillie, J., Bain, M., and Sinclair, J.H. (2002). Human cytomegalovirus infection inhibits epidermal growth factor (EGF) signalling by targeting EGF receptors. J. Gen. Virol. *83*, 2803–2810.

Fletcher, J.M., Prentice, H.G., and Grundy, J.E. (1998). Natural killer cell lysis of cytomegalovirus (CMV)-infected cells correlates with virally induced changes in cell surface lymphocyte function-associated antigen-3 (LFA-3) expression and not with the CMV-induced down-regulation of cell surface class I HLA. J. Immunol. *161*, 2365–2374.

Fraile-Ramos, A., Pelchen-Matthews, A., Kledal, T.N., Browne, H., Schwartz, T.W., and Marsh, M. (2002). Localization of HCMV UL33 and US27 in endocytic compartments and viral membranes. Traffic *3*, 218–232.

Gabaev, I., Steinbrück, L., Pokoyski, C., Pich, A., Stanton, R.J., Schwinzer, R., Schulz, T.F., Jacobs, R., Messerle, M., and Kay-Fedorov, P.C. (2011). The human cytomegalovirus UL11 protein interacts with the receptor tyrosine phosphatase CD45, resulting in functional paralysis of T cells. PLoS Pathog. *7*, e1002432.

Gao, J.L., and Murphy, P.M. (1994). Human cytomegalovirus open reading frame US28 encodes a functional beta chemokine receptor. J. Biol. Chem. *269*, 28539–28542.

Garlanda, C., Dinarello, C.A., and Mantovani, A. (2013). The interleukin-1 family: back to the future. Immunity *39*, 1003–1018.

Goldmacher, V.S., Bartle, L.M., Skaletskaya, A., Dionne, C.A., Kedersha, N.L., Vater, C.A., Han, J.W., Lutz, R.J., Watanabe, S., Cahir McFarland, E.D., et al. (1999). A cytomegalovirus-encoded mitochondria-localized inhibitor of apoptosis structurally unrelated to Bcl-2. Proc. Natl. Acad. Sci. USA *96*, 12536–12541.

Goujon, C., Moncorgé, O., Bauby, H., Doyle, T., Ward, C.C., Schaller, T., Hué, S., Barclay, W.S., Schulz, R., and Malim, M.H. (2013). Human MX2 is an interferon-induced post-entry inhibitor of HIV-1 infection. Nature *502*, 559–562.

Grainger, L., Cicchini, L., Rak, M., Petrucelli, A., Fitzgerald, K.D., Semler, B.L., and Goodrum, F. (2010). Stress-inducible alternative translation initiation of human cytomegalovirus latency protein pUL138. J. Virol. *84*, 9472–9486.

Gredmark, S., Strååt, K., Homman-Loudiyi, M., Kannisto, K., and Söderberg-Nauclér, C. (2007). Human cytomegalovirus downregulates expression of receptors for platelet-derived growth factor by smooth muscle cells. J. Virol. *81*, 5112–5120.

Greis, K.D., Gibson, W., and Hart, G.W. (1994). Site-specific glycosylation of the human cytomegalovirus tegument basic phosphoprotein (UL32) at serine 921 and serine 952. J. Virol. *68*, 8339–8349.

Grundy, J.E., and Downes, K.L. (1993). Up-regulation of LFA-3 and ICAM-1 on the surface of fibroblasts infected with cytomegalovirus. Immunology *78*, 405–412.

Hengel, H., Koopmann, J.O., Flohr, T., Muranyi, W., Goulmy, E., Hämmerling, G.J., Koszinowski, U.H., and Momburg, F. (1997). A viral ER-resident glycoprotein inactivates the MHC-encoded peptide transporter. Immunity *6*, 623–632.

Hewitt, E.W., Gupta, S.S., and Lehner, P.J. (2001). The human cytomegalovirus gene product US6 inhibits ATP binding by TAP. EMBO J. *20*, 387–396.

Hitomi, S., Kozuka-Hata, H., Chen, Z., Sugano, S., Yamaguchi, N., and Watanabe, S. (1997). Human cytomegalovirus open reading frame UL11 encodes a highly polymorphic protein expressed on the infected cell surface. Arch. Virol. *142*, 1407–1427.

Huber, M.T., and Compton, T. (1999). Intracellular formation and processing of the heterotrimeric gH-gL-gO (gCIII) glycoprotein envelope complex of human cytomegalovirus. J. Virol. *73*, 3886–3892.

Huber, M.T., Tomazin, R., Wisner, T., Boname, J., and Johnson, D.C. (2002). Human cytomegalovirus US7, US8, US9, and US10 are cytoplasmic glycoproteins, not found at cell surfaces, and US9 does not mediate cell-to-cell spread. J. Virol. *76*, 5748–5758.

Hwang, J., Saffert, R.T., and Kalejta, R.F. (2011). Elongin B-mediated epigenetic alteration of viral chromatin correlates with efficient human cytomegalovirus gene expression and replication. MBio *2*, e00023–e11.

Johnson, R.A., Huong, S.M., and Huang, E.S. (2000). Activation of the mitogen-activated protein kinase p38 by human cytomegalovirus infection through two distinct pathways: a novel mechanism for activation of p38. J. Virol. *74*, 1158–1167.

Kamil, J.P., Hume, A.J., Jurak, I., Münger, K., Kalejta, R.F., and Coen, D.M. (2009). Human papillomavirus 16 E7 inactivator of retinoblastoma family proteins complements human cytomegalovirus lacking UL97 protein kinase. Proc. Natl. Acad. Sci. USA *106*, 16823–16828.

Kanehisa, M., and Goto, S. (2000). KEGG: kyoto encyclopedia of genes and genomes. Nucleic Acids Res. *28*, 27–30.

Karnowski, A., Chevrier, S., Belz, G.T., Mount, A., Emslie, D., D'Costa, K., Tarlinton, D.M., Kallies, A., and Corcoran, L.M. (2012). B and T cells collaborate in antiviral responses via IL-6, IL-21, and transcriptional activator and coactivator, Oct2 and OBF-1. J. Exp. Med. *209*, 2049–2064.

Kato, H., Takeuchi, O., Sato, S., Yoneyama, M., Yamamoto, M., Matsui, K., Uematsu, S., Jung, A., Kawai, T., Ishii, K.J., et al. (2006). Differential roles of MDA5 and RIG-I helicases in the recognition of RNA viruses. Nature *441*, 101–105.

Kaye, J., Browne, H., Stoffel, M., and Minson, T. (1992a). The UL16 gene of human cytomegalovirus encodes a glycoprotein that is dispensable for growth in vitro. J. Virol. *66*, 6609–6615.

Kaye, J.F., Gompels, U.A., and Minson, A.C. (1992b). Glycoprotein H of human cytomegalovirus (HCMV) forms a stable complex with the HCMV UL115 gene product. J. Gen. Virol. *73*, 2693–2698.

Kiefel, H., Bondong, S., Hazin, J., Ridinger, J., Schirmer, U., Riedle, S., and Altevogt, P. (2012). L1CAM: a major driver for tumor cell invasion and motility. Cell Adhes. Migr. *6*, 374–384.

Kim, S.Y., Yasuda, S., Tanaka, H., Yamagata, K., and Kim, H. (2011b). Non-clustered protocadherin. Cell Adhes. Migr. *5*, 97–105.

Kinzler, E.R., Theiler, R.N., and Compton, T. (2002). Expression and reconstitution of the gH/gL/gO complex of human cytomegalovirus. J. Clin. Virol. *25* (*Suppl 2*), S87–S95.

Kolesnikova, T.V., Stipp, C.S., Rao, R.M., Lane, W.S., Luscinskas, F.W., and Hemler, M.E. (2004). EWI-2 modulates lymphocyte integrin alpha4beta1 functions. Blood *103*, 3013–3019.

Kropff, B., Koedel, Y., Britt, W., and Mach, M. (2010). Optimal replication of human cytomegalovirus correlates with endocytosis of glycoprotein gpUL132. J. Virol. *84*, 7039–7052.

Kuja-Panula, J., Kiiltomäki, M., Yamashiro, T., Rouhiainen, A., and Rauvala, H. (2003). AMIGO, a transmembrane protein implicated in axon tract development, defines a novel protein family with leucine-rich repeats. J. Cell Biol. *160*, 963–973.

LaPierre, L.A., and Biegalke, B.J. (2001). Identification of a novel transcriptional repressor encoded by human cytomegalovirus. J. Virol. *75*, 6062–6069.

Leach, F.S., and Mocarski, E.S. (1989). Regulation of cytomegalovirus late-gene expression: differential use of three start sites in the transcriptional activation of ICP36 gene expression. J. Virol. *63*, 1783–1791.

Lee, J., Koh, K., Kim, Y.E., Ahn, J.H., and Kim, S. (2013). Upregulation of Nrf2 expression by human cytomegalovirus infection protects host cells from oxidative stress. J. Gen. Virol. *94*, 1658–1668.

Lehner, P.J., Karttunen, J.T., Wilkinson, G.W., and Cresswell, P. (1997). The human cytomegalovirus US6 glycoprotein inhibits transporter associated with antigen processing-dependent peptide translocation. Proc. Natl. Acad. Sci. USA *94*, 6904–6909.

Lilley, B.N., Ploegh, H.L., and Tirabassi, R.S. (2001). Human cytomegalovirus open reading frame TRL11/IRL11 encodes an immunoglobulin G Fc-binding protein. J. Virol. *75*, 11218–11221.

Mach, M., Kropff, B., Dal Monte, P., and Britt, W. (2000). Complex formation by human cytomegalovirus glycoproteins M (gpUL100) and N (gpUL73). J. Virol. *74*, 11881–11892.

Maidji, E., Tugizov, S., Abenes, G., Jones, T., and Pereira, L. (1998). A novel human cytomegalovirus glycoprotein, gpUS9, which promotes cell-to-cell spread in polarized epithelial cells, colocalizes with the cytoskeletal proteins E-cadherin and F-actin. J. Virol. *72*, 5717–5727.

Makarov, A., and Denisov, E. (2009). Dynamics of ions of intact proteins in the Orbitrap mass analyzer. J. Am. Soc. Mass Spectrom. *20*, 1486–1495.

Margulies, B.J., and Gibson, W. (2007). The chemokine receptor homologue encoded by US27 of human cytomegalovirus is heavily glycosylated and is present in infected human foreskin fibroblasts and enveloped virus particles. Virus Res. *123*, 57–71.

Margulies, B.J., Browne, H., and Gibson, W. (1996). Identification of the human cytomegalovirus G protein-coupled receptor homologue encoded by UL33 in infected cells and enveloped virus particles. Virology *225*, 111–125.

Marshall, E.E., Bierle, C.J., Brune, W., and Geballe, A.P. (2009). Essential role for either TRS1 or IRS1 in human cytomegalovirus replication. J. Virol. *83*, 4112–4120.

McCormick, A.L., Roback, L., Livingston-Rosanoff, D., and St Clair, C. (2010). The human cytomegalovirus UL36 gene controls caspase-dependent and -independent cell death programs activated by infection of monocytes differentiating to macrophages. J. Virol. *84*, 5108–5123.

Michel, D., Milotić, I., Wagner, M., Vaida, B., Holl, J., Ansorge, R., and Mertens, T. (2005). The human cytomegalovirus UL78 gene is highly conserved among clinical isolates, but is dispensable for replication in fibroblasts and a renal artery organ-culture system. J. Gen. Virol. *86*, 297–306.

Montag, C., Wagner, J.A., Gruska, I., Vetter, B., Wiebusch, L., and Hagemeier, C. (2011). The latency-associated UL138 gene product of human cytomegalovirus sensitizes cells to tumor necrosis factor alpha (TNF-alpha) signaling by upregulating TNF-alpha receptor 1 cell surface expression. J. Virol. *85*, 11409–11421.

Mootha, V.K., Lindgren, C.M., Eriksson, K.F., Subramanian, A., Sihag, S., Lehar, J., Puigserver, P., Carlsson, E., Ridderstråle, M., Laurila, E., et al. (2003). PGC-1alpha-responsive genes involved in oxidative phosphorylation are coordinately downregulated in human diabetes. Nat. Genet. *34*, 267–273.

Morales, D.J., and Lenschow, D.J. (2013). The antiviral activities of ISG15. J. Mol. Biol. *425*, 4995–5008.

Nachmani, D., Lankry, D., Wolf, D.G., and Mandelboim, O. (2010). The human cytomegalovirus microRNA miR-UL112 acts synergistically with a cellular microRNA to escape immune elimination. Nat. Immunol. *11*, 806–813.

Nagaishi, T., Iijima, H., Nakajima, A., Chen, D., and Blumberg, R.S. (2006). Role of CEACAM1 as a regulator of T cells. Ann. N Y Acad. Sci. *1072*, 155–175.

Naik, U.P., and Eckfeld, K. (2003). Junctional adhesion molecule 1 (JAM-1). J. Biol. Regul. Homeost. Agents *17*, 341–347.

Neote, K., DiGregorio, D., Mak, J.Y., Horuk, R., and Schall, T.J. (1993). Molecular cloning, functional expression, and signaling characteristics of a C-C chemokine receptor. Cell *72*, 415–425.

O'Connor, C.M., and Shenk, T. (2012). Human cytomegalovirus pUL78 G protein-coupled receptor homologue is required for timely cell entry in epithelial cells but not fibroblasts. J. Virol. *86*, 11425–11433.

Oudshoorn, D., van Boheemen, S., Sánchez-Aparicio, M.T., Rajsbaum, R., García-Sastre, A., and Versteeg, G.A. (2012). HERC6 is the main E3 ligase for global ISG15 conjugation in mouse cells. PLoS ONE *7*, e29870.

Park, M.Y., Kim, Y.E., Seo, M.R., Lee, J.R., Lee, C.H., and Ahn, J.H. (2006). Interactions among four proteins encoded by the human cytomegalovirus UL112-113 region regulate their intranuclear targeting and the recruitment of UL44 to prereplication foci. J. Virol. *80*, 2718–2727.

Park, B., Spooner, E., Houser, B.L., Strominger, J.L., and Ploegh, H.L. (2010). The HCMV membrane glycoprotein US10 selectively targets HLA-G for degradation. J. Exp. Med. *207*, 2033–2041.

Peisley, A., and Hur, S. (2013). Multi-level regulation of cellular recognition of viral dsRNA. Cell. Mol. Life Sci. *70*, 1949–1963.

Penfold, M.E., Dairaghi, D.J., Duke, G.M., Saederup, N., Mocarski, E.S., Kemble, G.W., and Schall, T.J. (1999). Cytomegalovirus encodes a potent alpha chemokine. Proc. Natl. Acad. Sci. USA *96*, 9839–9844.

Pereira, L., Maidji, E., Tugizov, S., and Jones, T. (1995). Deletion mutants in human cytomegalovirus glycoprotein US9 are impaired in cell-cell transmission and in altering tight junctions of polarized human retinal pigment epithelial cells. Scand. J. Infect. Dis. Suppl. *99*, 82–87.

Phillips, S.L., and Bresnahan, W.A. (2012). The human cytomegalovirus (HCMV) tegument protein UL94 is essential for secondary envelopment of HCMV virions. J. Virol. *86*, 2523–2532.

Phillips, A.J., Tomasec, P., Wang, E.C., Wilkinson, G.W., and Borysiewicz, L.K. (1998). Human cytomegalovirus infection downregulates expression of the cellular aminopeptidases CD10 and CD13. Virology *250*, 350–358.

Pleskoff, O., Tréboute, C., Brelot, A., Heveker, N., Seman, M., and Alizon, M. (1997). Identification of a chemokine receptor encoded by human cytomegalovirus as a cofactor for HIV-1 entry. Science *276*, 1874–1878.

Pleskoff, O., Tréboute, C., and Alizon, M. (1998). The cytomegalovirus-encoded chemokine receptor US28 can enhance cell-cell fusion mediated by different viral proteins. J. Virol. *72*, 6389–6397.

Prod'homme, V., Griffin, C., Aicheler, R.J., Wang, E.C., McSharry, B.P., Rickards, C.R., Stanton, R.J., Borysiewicz, L.K., López-Botet, M., Wilkinson, G.W., and Tomasec, P. (2007). The human cytomegalovirus MHC class I homolog UL18 inhibits LIR-1+ but activates LIR-1- NK cells. J. Immunol. *178*, 4473–4481.

Prod'homme, V., Tomasec, P., Cunningham, C., Lemberg, M.K., Stanton, R.J., McSharry, B.P., Wang, E.C., Cuff, S., Martoglio, B., Davison, A.J., et al. (2012). Human cytomegalovirus UL40 signal peptide regulates cell surface expression of the NK cell ligands HLA-E and gpUL18. J. Immunol. *188*, 2794–2804.

Radsak, K., Eickmann, M., Mockenhaupt, T., Bogner, E., Kern, H., Eis-Hübinger, A., and Reschke, M. (1996). Retrieval of human cytomegalovirus glycoprotein B from the infected cell surface for virus envelopment. Arch. Virol. *141*, 557–572.

Rao, T.P., and Kühl, M. (2010). An updated overview on Wnt signaling pathways: a prelude for more. Circ. Res. *106*, 1798–1806.

Rappsilber, J., Mann, M., and Ishihama, Y. (2007). Protocol for micro-purification, enrichment, pre-fractionation and storage of peptides for proteomics using StageTips. Nat. Protoc. *2*, 1896–1906.

Raschperger, E., Engstrom, U., Pettersson, R.F., and Fuxe, J. (2004). CLMP, a novel member of the CTX family and a new component of epithelial tight junctions. J. Biol. Chem. *279*, 796–804.

Rebhan, M., Chalifa-Caspi, V., Prilusky, J., and Lancet, D. (1997). GeneCards: integrating information about genes, proteins and diseases. Trends Genet. *13*, 163.

Reim, N.I., Kamil, J.P., Wang, D., Lin, A., Sharma, M., Ericsson, M., Pesola, J.M., Golan, D.E., and Coen, D.M. (2013). Inactivation of retinoblastoma protein does not overcome the requirement for human cytomegalovirus UL97 in lamina disruption and nuclear egress. J. Virol. *87*, 5019–5027.

Rölle, A., Mousavi-Jazi, M., Eriksson, M., Odeberg, J., Söderberg-Nauclér, C., Cosman, D., Kärre, K., and Cerboni, C. (2003). Effects of human cytomegalovirus infection on ligands for the activating NKG2D receptor of NK cells: up-regulation of UL16-binding protein (ULBP)1 and ULBP2 is counteracted by the viral UL16 protein. J. Immunol. *171*, 902–908.

Romanowski, M.J., Garrido-Guerrero, E., and Shenk, T. (1997). pIRS1 and pTRS1 are present in human cytomegalovirus virions. J. Virol. *71*, 5703–5705.

Savaryn, J.P., Reitsma, J.M., Bigley, T.M., Halligan, B.D., Qian, Z., Yu, D., and Terhune, S.S. (2013). Human cytomegalovirus pUL29/28 and pUL38 repression of p53-regulated p21CIP1 and caspase 1 promoters during infection. J. Virol. *87*, 2463–2474.

Sharma, A., Verhaagen, J., and Harvey, A.R. (2012). Receptor complexes for each of the Class 3 Semaphorins. Front Cell Neurosci *6*, 28.

Shi-Chen Ou, D., Lee, S.B., Chu, C.S., Chang, L.H., Chung, B.C., and Juan, L.J. (2011). Transcriptional activation of endoplasmic reticulum chaperone GRP78 by HCMV IE1-72 protein. Cell Res. *21*, 642–653.

Shikhagaie, M., Mercé-Maldonado, E., Isern, E., Muntasell, A., Albà, M.M., López-Botet, M., Hengel, H., and Angulo, A. (2012). The human cytomegalovirus-specific UL1 gene encodes a late-phase glycoprotein incorporated in the virion envelope. J. Virol. *86*, 4091–4101.

Silva, M.C., Yu, Q.C., Enquist, L., and Shenk, T. (2003). Human cytomegalovirus UL99-encoded pp28 is required for the cytoplasmic envelopment of tegument-associated capsids. J. Virol. *77*, 10594–10605.

Skaletskaya, A., Bartle, L.M., Chittenden, T., McCormick, A.L., Mocarski, E.S., and Goldmacher, V.S. (2001). A cytomegalovirus-encoded inhibitor of apoptosis that suppresses caspase-8 activation. Proc. Natl. Acad. Sci. USA *98*, 7829–7834.

Sleeman, M.W., Anderson, K.D., Lambert, P.D., Yancopoulos, G.D., and Wiegand, S.J. (2000). The ciliary neurotrophic factor and its receptor, CNTFR alpha. Pharm. Acta Helv. *74*, 265–272.

Smith, J.A., and Pari, G.S. (1995). Expression of human cytomegalovirus UL36 and UL37 genes is required for viral DNA replication. J. Virol. *69*, 1925–1931.

Smith, W., Tomasec, P., Aicheler, R., Loewendorf, A., Nemčovičová, I., Wang, E.C., Stanton, R.J., Macauley, M., Norris, P., Willen, L., et al. (2013). Human cytomegalovirus glycoprotein UL141 targets the TRAIL death receptors to thwart host innate antiviral defenses. Cell Host Microbe *13*, 324–335.

Spaderna, S., Blessing, H., Bogner, E., Britt, W., and Mach, M. (2002). Identification of glycoprotein gpTRL10 as a structural component of human cytomegalovirus. J. Virol. *76*, 1450–1460.

Spaderna, S., Hahn, G., and Mach, M. (2004). Glycoprotein gpTRL10 of human cytomegalovirus is dispensable for virus replication in human fibroblasts. Arch. Virol. *149*, 495–506.

Spaderna, S., Kropff, B., Ködel, Y., Shen, S., Coley, S., Lu, S., Britt, W., and Mach, M. (2005). Deletion of gpUL132, a structural component of human cytomegalovirus, results in impaired virus replication in fibroblasts. J. Virol. *79*, 11837–11847.

Spiller, O.B., Morgan, B.P., Tufaro, F., and Devine, D.V. (1996). Altered expression of host-encoded complement regulators on human cytomegalovirus-infected cells. Eur. J. Immunol. *26*, 1532–1538.

Stanton, R.J., Baluchova, K., Dargan, D.J., Cunningham, C., Sheehy, O., Seirafian, S., McSharry, B.P., Neale, M.L., Davies, J.A., Tomasec, P., et al. (2010). Reconstruction of the complete human cytomegalovirus genome in a BAC reveals RL13 to be a potent inhibitor of replication. J. Clin. Invest. *120*, 3191–3208.

Stern-Ginossar, N., Elefant, N., Zimmermann, A., Wolf, D.G., Saleh, N., Biton, M., Horwitz, E., Prokocimer, Z., Prichard, M., Hahn, G., et al. (2007). Host immune system gene targeting by a viral miRNA. Science *317*, 376–381.

Stern-Ginossar, N., Weisburd, B., Michalski, A., Le, V.T., Hein, M.Y., Huang, S.X., Ma, M., Shen, B., Qian, S.B., Hengel, H., et al. (2012). Decoding human cytomegalovirus. Science *338*, 1088–1093.

Strang, B.L., Geballe, A.P., and Coen, D.M. (2010). Association of human cytomegalovirus proteins IRS1 and TRS1 with the viral DNA polymerase accessory subunit UL44. J. Gen. Virol. *91*, 2167–2175.

Tadagaki, K., Tudor, D., Gbahou, F., Tschische, P., Waldhoer, M., Bomsel, M., Jockers, R., and Kamal, M. (2012). Human cytomegalovirus-encoded UL33 and UL78 heteromerize with host CCR5 and CXCR4 impairing their HIV coreceptor activity. Blood *119*, 4908–4918.

Tirabassi, R.S., and Ploegh, H.L. (2002). The human cytomegalovirus US8 glycoprotein binds to major histocompatibility complex class I products. J. Virol. *76*, 6832–6835.

Tomasec, P., Braud, V.M., Rickards, C., Powell, M.B., McSharry, B.P., Gadola, S., Cerundolo, V., Borysiewicz, L.K., McMichael, A.J., and Wilkinson, G.W. (2000). Surface expression of HLA-E, an inhibitor of natural killer cells, enhanced by human cytomegalovirus gpUL40. Science *287*, 1031.

Tran, K., Kamil, J.P., Coen, D.M., and Spector, D.H. (2010). Inactivation and disassembly of the anaphase-promoting complex during human cytomegalovirus infection is associated with degradation of the APC5 and APC4 subunits and does not require UL97-mediated phosphorylation of Cdh1. J. Virol. *84*, 10832–10843.

Tugizov, S., Navarro, D., Paz, P., Wang, Y., Qadri, I., and Pereira, L. (1994). Function of human cytomegalovirus glycoprotein B: syncytium formation in cells constitutively expressing gB is blocked by virus-neutralizing antibodies. Virology *201*, 263–276.

Umashankar, M., Petrucelli, A., Cicchini, L., Caposio, P., Kreklywich, C.N., Rak, M., Bughio, F., Goldman, D.C., Hamlin, K.L., Nelson, J.A., et al. (2011). A novel human cytomegalovirus locus modulates cell type-specific outcomes of infection. PLoS Pathog. *7*, e1002444.

Vahdati-Ben Arieh, S., Laham, N., Schechter, C., Yewdell, J.W., Coligan, J.E., and Ehrlich, R. (2003). A single viral protein HCMV US2 affects antigen presentation and intracellular iron homeostasis by degradation of classical HLA class I and HFE molecules. Blood *101*, 2858–2864.

Vanarsdall, A.L., Chase, M.C., and Johnson, D.C. (2011). Human cytomegalovirus glycoprotein gO complexes with gH/gL, promoting interference with viral entry into human fibroblasts but not entry into epithelial cells. J. Virol. *85*, 11638–11645.

Varnum, S.M., Streblow, D.N., Monroe, M.E., Smith, P., Auberry, K.J., Pasa-Tolic, L., Wang, D., Camp, D.G., 2nd, Rodland, K., Wiley, S., et al. (2004). Identification of proteins in human cytomegalovirus (HCMV) particles: the HCMV proteome. J. Virol. *78*, 10960–10966.

Viner, R., Bomgarden, R., Blank, R., and Rogers, J. (2013). Increasing the Multiplexing of Protein Quantitation from 6- to 10-Plex with Reporter Ion Isotopologues. http://apps.thermoscientific.com/media/cmd/ASMS-TNG-Roadshow/TNG/resouces/PN_ASMS13_W617_RViner.pdf.

Viswanathan, K., Smith, M.S., Malouli, D., Mansouri, M., Nelson, J.A., and Früh, K. (2011). BST2/Tetherin enhances entry of human cytomegalovirus. PLoS Pathog. *7*, e1002332.

Wagner, S., Arnold, F., Wu, Z., Schubert, A., Walliser, C., Tadagaki, K., Jockers, R., Mertens, T., and Michel, D. (2012). The 7-transmembrane protein homologue UL78 of the human cytomegalovirus forms oligomers and traffics between the plasma membrane and different intracellular compartments. Arch. Virol. *157*, 935–949.

Wang, S.K., Hu, C.H., Lu, M.C., Duh, C.Y., Liao, P.C., and Tyan, Y.C. (2009). Novel virus-associated proteins encoded by UL112-113 of human cytomegalovirus. J. Gen. Virol. *90*, 2840–2848.

Wang, N., Ma, Y., Li, M., Gao, S., Wang, L., Qi, Y., Ji, Y., Sun, Z., and Ruan, Q. (2013). Transcription characteristics of the human cytomegalovirus UL13 gene. Arch. Virol. *158*, 473–477.

Weekes, M.P., Antrobus, R., Lill, J.R., Duncan, L.M., Hör, S., and Lehner, P.J. (2010). Comparative analysis of techniques to purify plasma membrane proteins. J. Biomol. Tech. *21*, 108–115.

Wei, C.H., and Ryu, S.E. (2012). Homophilic interaction of the L1 family of cell adhesion molecules. Exp. Mol. Med. *44*, 413–423.

Welte, S.A., Sinzger, C., Lutz, S.Z., Singh-Jasuja, H., Sampaio, K.L., Eknigk, U., Rammensee, H.G., and Steinle, A. (2003). Selective intracellular retention of virally induced NKG2D ligands by the human cytomegalovirus UL16 glycoprotein. Eur. J. Immunol. *33*, 194–203.

Wills, M.R., Ashiru, O., Reeves, M.B., Okecha, G., Trowsdale, J., Tomasec, P., Wilkinson, G.W., Sinclair, J., and Sissons, J.G. (2005). Human cytomegalovirus encodes an MHC class I-like molecule (UL142) that functions to inhibit NK cell lysis. J. Immunol. *175*, 7457–7465.

Wong, J.J., Pung, Y.F., Sze, N.S., and Chin, K.C. (2006). HERC5 is an IFN-induced HECT-type E3 protein ligase that mediates type I IFN-induced ISGylation of protein targets. Proc. Natl. Acad. Sci. USA *103*, 10735–10740.

Yagi, T. (2008). Clustered protocadherin family. Dev. Growth Differ. *50* (*Suppl 1*), S131–S140.

Yu, Y., Maguire, T.G., and Alwine, J.C. (2011). Human cytomegalovirus activates glucose transporter 4 expression to increase glucose uptake during infection. J. Virol. *85*, 1573–1580.

Yu, Y., Maguire, T.G., and Alwine, J.C. (2012). Human cytomegalovirus infection induces adipocyte-like lipogenesis through activation of sterol regulatory element binding protein 1. J. Virol. *86*, 2942–2949.

Yurochko, A.D., Mayo, M.W., Poma, E.E., Baldwin, A.S., Jr., and Huang, E.S. (1997). Induction of the transcription factor Sp1 during human cytomegalovirus infection mediates upregulation of the p65 and p105/p50 NF-kappaB promoters. J. Virol. *71*, 4638–4648.

Zhang, A., Williamson, C.D., Wong, D.S., Bullough, M.D., Brown, K.J., Hathout, Y., and Colberg-Poley, A.M. (2011). Quantitative proteomic analyses of human cytomegalovirus-induced restructuring of endoplasmic reticulum-mitochondrial contacts at late times of infection. Mol. Cell. Proteomics *10*, M111 009936.

Zhou, X., Michal, J.J., Zhang, L., Ding, B., Lunney, J.K., Liu, B., and Jiang, Z. (2013). Interferon induced IFIT family genes in host antiviral defense. Int. J. Biol. Sci. *9*, 200–208.

Zhu, H., Cong, J.P., Yu, D., Bresnahan, W.A., and Shenk, T.E. (2002). Inhibition of cyclooxygenase 2 blocks human cytomegalovirus replication. Proc. Natl. Acad. Sci. USA *99*, 3932–3937.

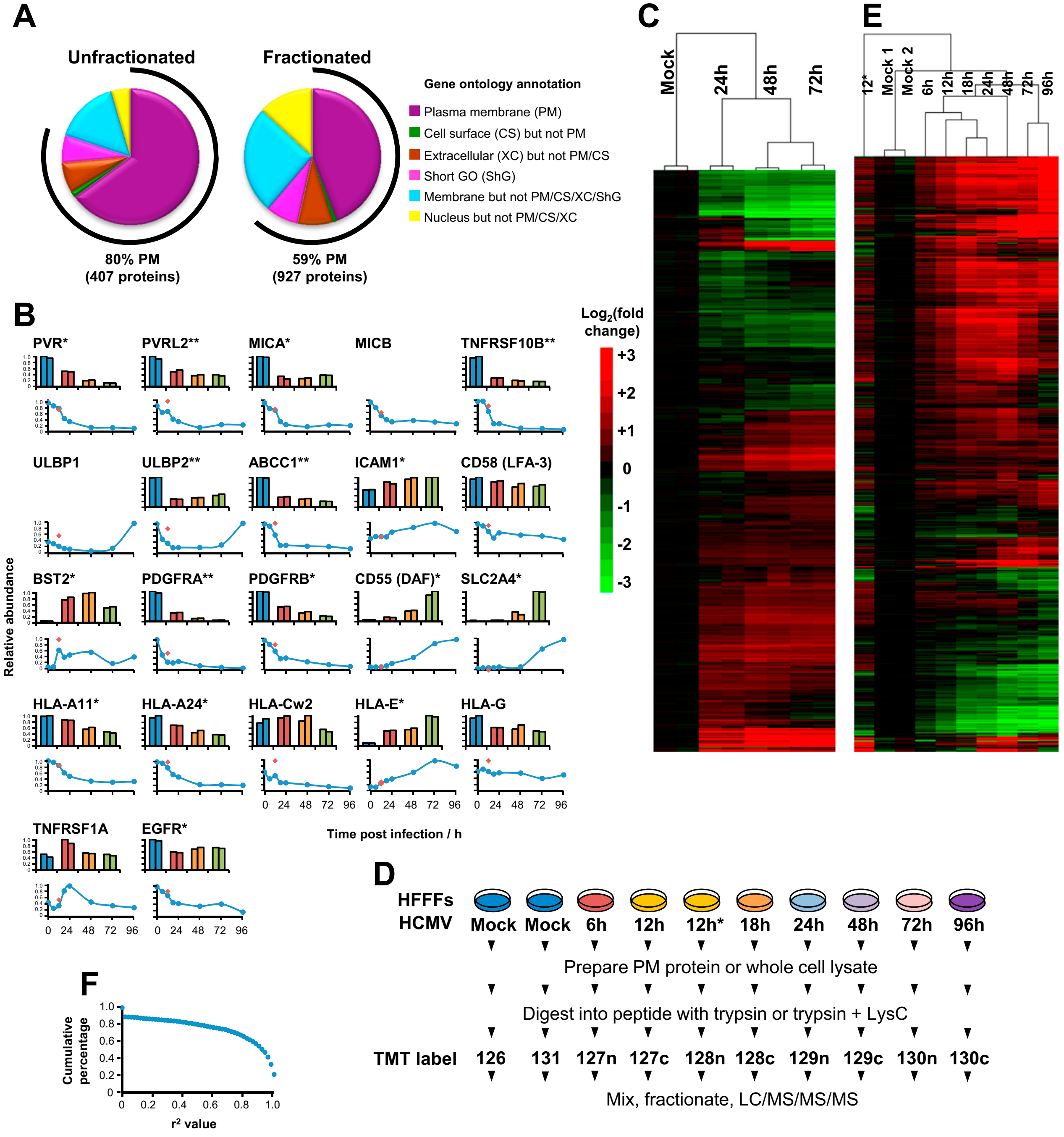

Figure S1. Temporal Plasma Membrane Profiling of HCMV-Infected Fibroblasts, Related to Figure 1

(A) Gene ontology annotation of proteins quantified in experiment PM1. Peptides were analyzed by mass spectrometry either unfractionated, or after division into 6 fractions using offline strong cation exchange. ''Short GO'' refers to a subset of proteins annotated by GO as integral to the membrane, but with no subcellular assignment and a short 4- or 5-part GO cellular compartment term (Weekes et al., 2012).

(B) Quantitation of all cell surface proteins that exhibit previously reported changes during productive HCMV infection in HFFFs. Protein temporal profiles correlated well between repeat time courses PM1 (upper panels) and PM2 (lower panels). MICB and ULBP1 were not quantified in experiment PM1. Because of sequence similarities it was not possible to distinguish individual isoforms of HLA-B, and these data are not included. Red diamonds – irradiated HCMV infection at 12h. One-way ANOVA with multiple hypothesis correction: *p < 0.005, **p < 0.0001. Red diamonds – 12h after infection with irradiated HCMV.

(C) Hierarchical clustering of proteins quantified in whole-cell lysate experiment WCL1. Fold change was limited to a maximum of 50.

(D) Workflow of 10-plex TMT experiments. *12h after infection with irradiated HCMV.

(E) Hierarchical clustering of proteins quantified in plasma membrane experiment PM2. Fold change was limited to a maximum of 50.

(F) Correlation between proteins quantified in both experiments WCL1 and WCL2.

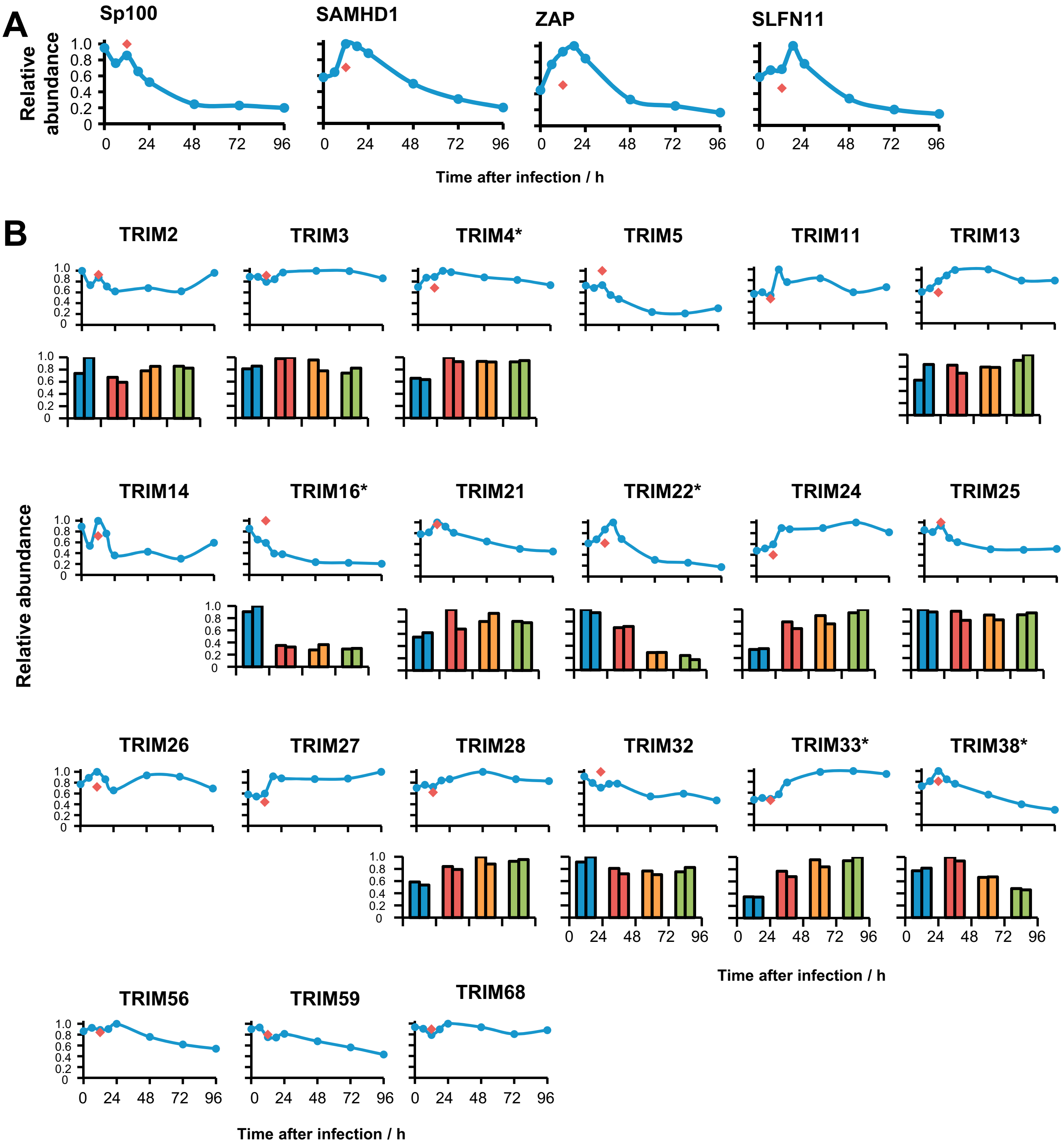

Figure S2. Modulation of Viral Restriction Factors during HCMV Infection, Related to Figure 2

(A) Sp100 is known to be targeted for degradation by HCMV IE1 (Kim et al., 2011a). Modulation of SAMHD1, ZAP and SLFN11 had not previously been recognized.

(B) Quantitation of 21 TRIMs during infection. Results are shown from experiments WCL2 (top sets of panels) and WCL1 (bottom sets of panels). 8 TRIMs were not quantified in experiment WCL1. One-way ANOVA with multiple hypothesis correction (experiment WCL1): *$p < 0.005$.

Red diamonds – 12h after infection with irradiated HCMV.

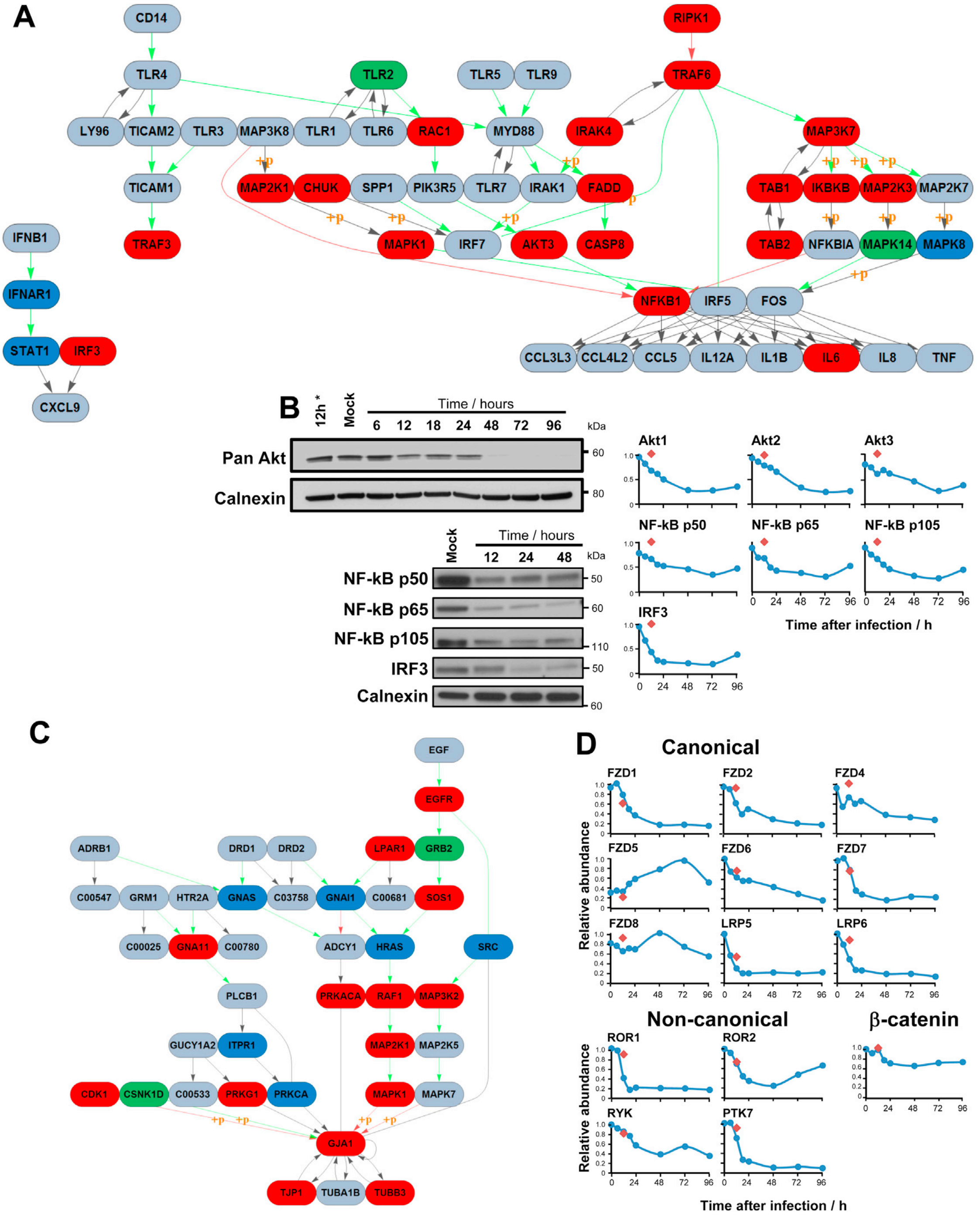

Figure S3. QTV Provides Insights into Modulated Signaling Pathways, Related to Figure 3

(A) The Toll-like receptor signaling pathway and its modulation during HCMV infection, based on the KEGG pathway (Kanehisa and Goto, 2000). Proteins (ovals) are shaded to correspond to their clusters (Figure 3B): red (downregulated), blue (unchanged), green (upregulated), gray (not quantified). Edges (from KEGG) are shaded green with arrows, to indicate that protein A activates protein B and pink to indicate that protein A inhibits protein B. A single gray/black edge with arrow indicates that protein A affects protein B - not labeled as activation or inhibition. A paired gray/black arrow between proteins A and B: protein A and B bind to or are associated with one another. Phosphorylation events are labeled with p+.

(B) Immunoblots of HFFF infected with HCMV confirm proteomic profiles for three TLR-pathway members.

(C) The gap junction pathway, shaded as for Figure S3A.

(D) Modulation of cell surface wnt receptors. 11/13 quantified canonical and non-canonical wnt receptors were downregulated. The WCL2 β-catenin profile demonstrated a modest decline over time. Red diamonds – 12h after infection with irradiated HCMV.

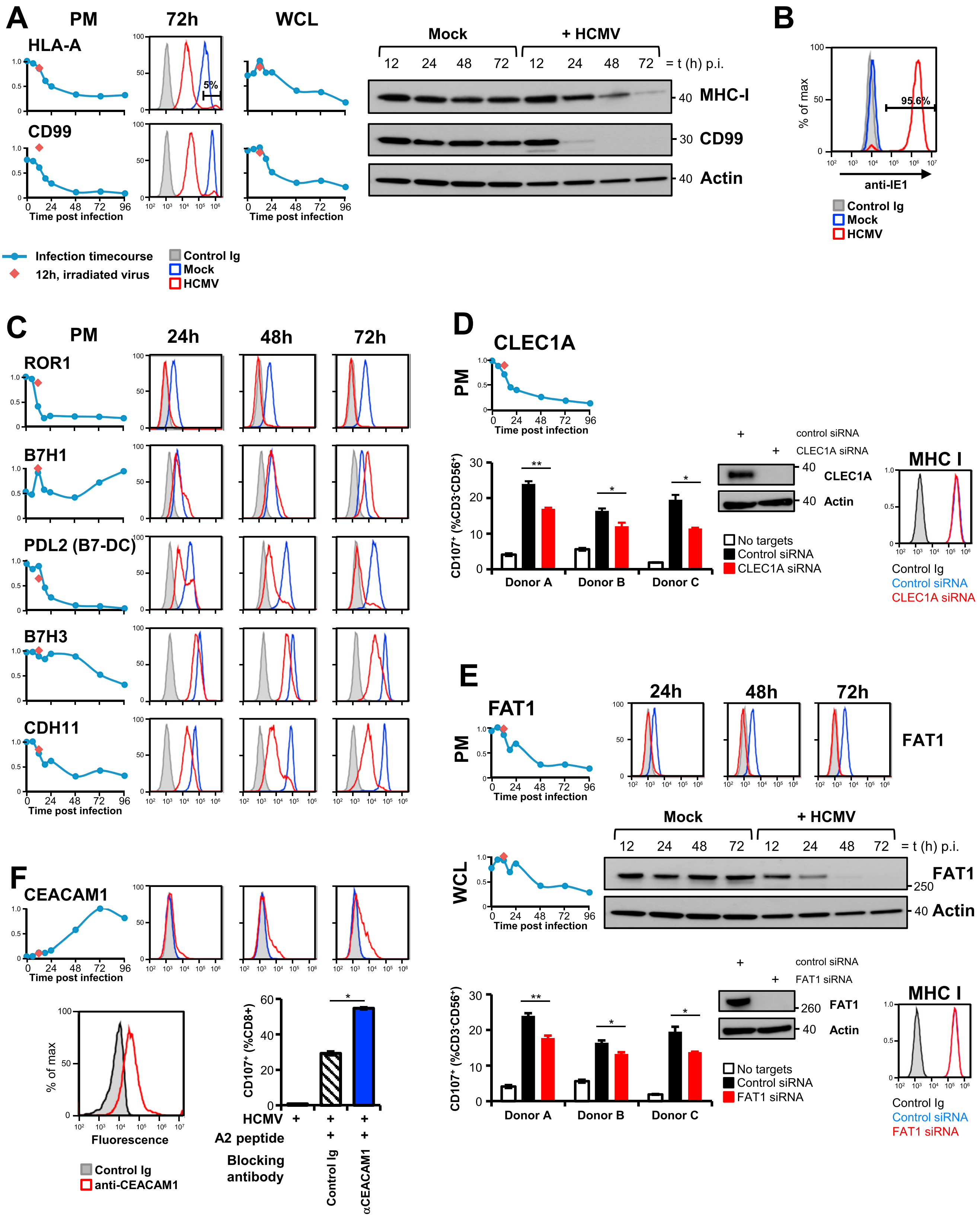

(legend on next page)

Figure S4. Validation of Temporal Profiles by Flow Cytometry and Immunoblot and Investigation of Novel Immune Ligands, Related to Figure 4

(A) PM and WCL profiles of MHC-I (HLA-A11) and CD99, validated by flow cytometry and immunoblot. $\geq$ 95% of HFFF were routinely infected by HCMV, assessed by flow cytometry for cell surface MHC-I.

(B) Representative intracellular flow cytometry of 24h-infected HFFF with anti-IE1 confirms > 95% infection efficiency.

(C) Flow cytometry of HFFF infected with HCMV confirms proteomic profiles for five additional cell surface proteins.

(D and E) NK degranulation assays suggest that CLEC1A and FAT1 are novel activating NK ligands. Top panels – validation of temporal PM and WCL profiles by flow cytometry and immunoblot. CLEC1A was not quantified in any WCL QTV experiments but accumulated by immunoblot of whole-cell lysates, while depleting from the PM. Bottom panels – target cells underwent siRNA knockdown of CLEC1A (D) or FAT1 (E) and were then incubated with stimulated polyclonal NK cells from each of three donors. Degranulation of NK cells in response to both CLEC1A and FAT1 knockdown targets was significantly reduced compared to control. Error bars: +/− SEM (donors A, B), +/− range (donor C). * two-tailed p-value < 0.05, ** two-tailed p-value < 0.005. Cell surface MHC-I was unaffected by siRNA (right bottom panels, staining with W6-32 antibody or control Ig).

(F) CD8+ T cell degranulation assay suggests that CEACAM1 is a novel inhibitory ligand for CMV-specific cytotoxic T cells. Top panel – validation of temporal PM profile by flow cytometry. Bottom panels (left) – flow cytometry of a CD8+ T cell line specific to the HCMV HLA-A2 restricted IE1 peptide VLEETSVML confirmed CEACAM1 surface expression. (right) HCMV peptide-specific CD8+ effector cells were incubated with autologous fibroblasts that had been infected with HCMV for 72h then pulsed with peptide or left unpulsed. Effectors and targets were treated with control Ig or anti-CEACAM1. HCMV-specific T cell degranulation was significantly increased with CEACAM1 block. Error bars ± SEM. * two-tailed p-value < 0.0001.

y-axes of QTV plots represent relative abundance, and y-axes of flow cytometry plots represent % of max.

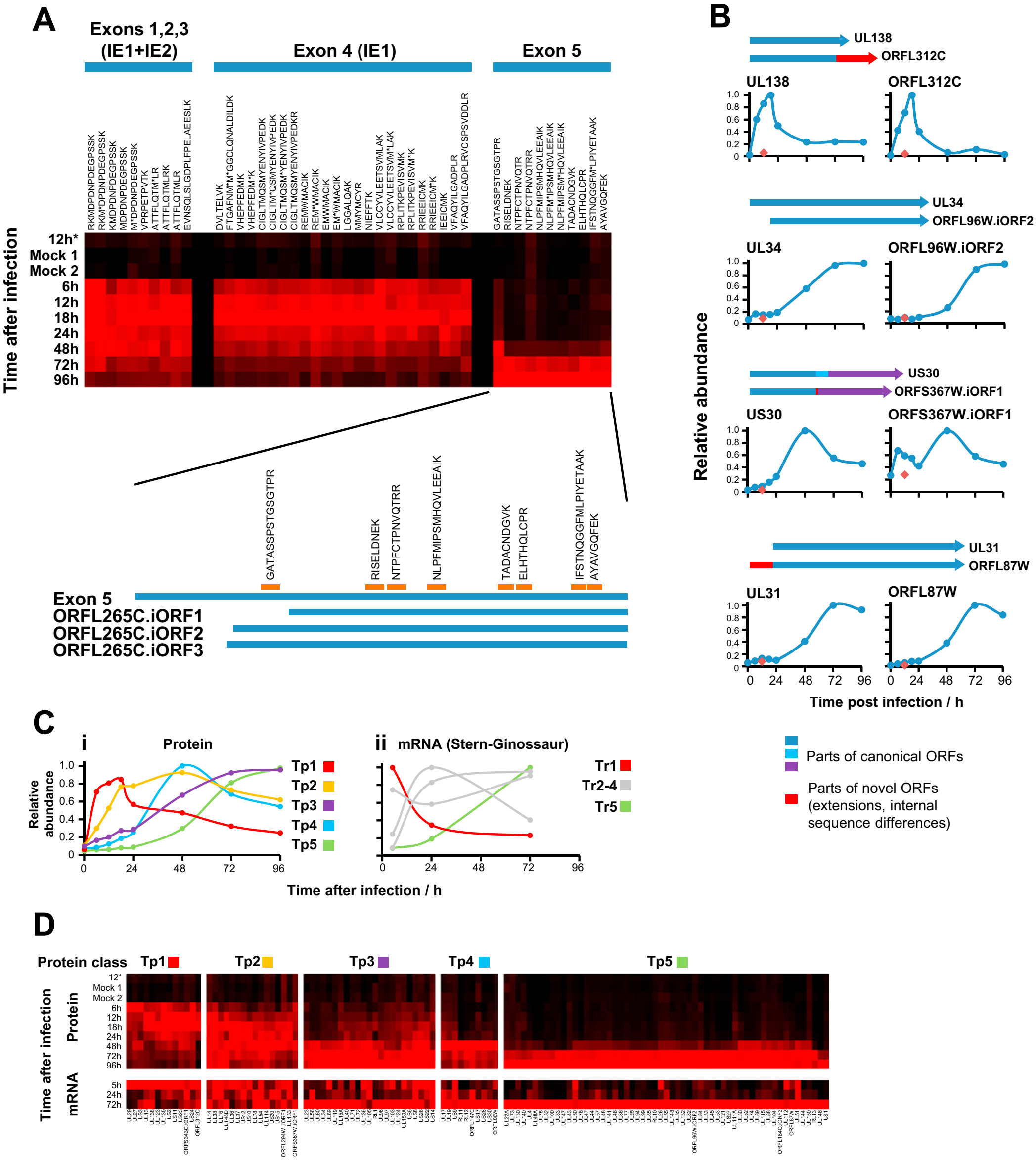

Figure S5. Further Details of Specific HCMV Proteins and Protein/mRNA Profiles, Related to Figure 5

(A) All peptides quantified from the major immediate early region spanning UL122 (IE2) – UL123 (IE1). Peptides from exon 4 are unique to UL123. Peptides from exons 1-3 were assigned to IE1 protein by our data processing software, according to the principles of parsimony. Expression of ten exon 5 peptides corresponding to ORFL265C.iORF1 (lower panel) peaked late at 96h, in comparison to a single peptide N-terminal to this region, which is likely to have derived from UL122 itself. The lower panel shows a map of internal ORFs detected by exon 5 ribosomal footprinting (Stern-Ginossar et al., 2012). * in peptide sequence: methionine oxidation.

(B) Relationship between four novel ORFs and their canonical HCMV counterparts, with temporal profiles. Each of the novel ORFs were quantified based only on unique peptides that could only have originated from that ORF. Peptides that could either have originated from the canonical protein or the novel ORF were assigned to the canonical protein.

(C) k-means clusters of (i) all quantified canonical HCMV proteins with 9 novel ORFs (experiment WCL2, also shown in Figure 5A) and (ii) all quantified canonical HCMV mRNAs and the same 9 novel ORFs (Stern-Ginossar et al., 2012). 5 clusters were selected in each case. The plots shown represent the average temporal profile for each cluster. As there were no intermediate mRNA time points between 5 and 24 hr, or between 24 and 72 hr, there was insufficient information to make an accurate comparison between the central three mRNA clusters and our Tp2, Tp3, or Tp4 class proteins. We therefore used mRNA data to define 3 classes: Tr1, Tr2-4, and Tr5. See Table S6C for details of the class of each protein.

(D) Comparison between temporal protein profiles (this study) and mRNA expression profiles (Stern-Ginossar et al., 2012), grouped according to protein class. The 134 viral genes quantified in both studies are shown. For each protein or transcript, expression was normalized to the maximum across the measured time points. There was extremely good correspondence between protein and mRNA temporal profiles for Tp1 and Tp5 protein classes. Correspondence was less good for Tp2-4 protein classes as there were insufficient intermediate RNA time points to determine when maximal mRNA expression occurred.

Red diamonds – 12h after infection with irradiated HCMV.

Cell

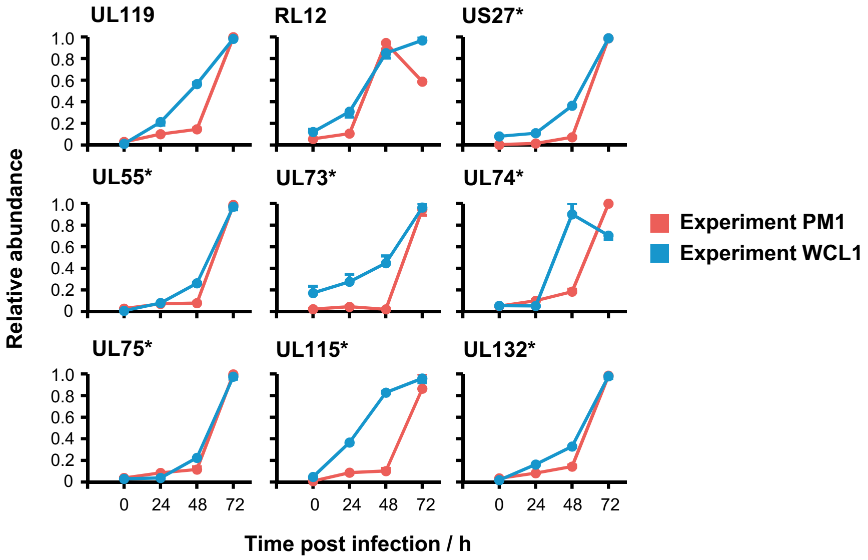

Figure S6. Temporal Profiles of "High-Confidence" Viral PM Proteins that Were Quantified in Experiment PM1, Related to Figure 6

Known virion envelope glycoproteins (starred) were generally detected significantly earlier in whole-cell lysates than in plasma membrane samples (Figure 6). Values shown are averages of two biological replicates, +/− range. See also Table S7.

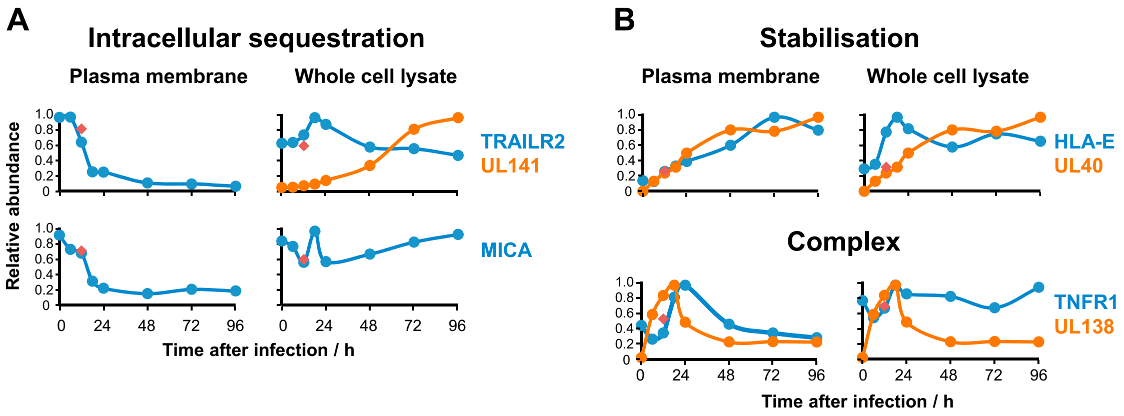

Figure S7. QTV Informs about Mechanism of Modulation of Cell-Surface Targets, Related to Figure 7

(A) Further examples of mechanistic insights into downregulated cell surface immunomodulators. TRAILR2 is retained in the ER by UL141 (Smith et al., 2013). MICA is retained in the *cis*-Golgi by UL142 (Ashiru et al., 2009). UL142 was only quantified at the plasma membrane (Figure S5A) and is not shown here.

(B) Examples of mechanistic insights into upregulated cell surface ligands, or receptors with complex patterns of expression. Signal peptide from HCMV UL40 acts to promote cell surface expression of HLA-E (Prod'homme et al., 2012). The WCL pattern of expression of UL40 was extremely similar to cell surface HLA-E, and is shown overlying both PM and WCL data to illustrate this. TNFR1 cell surface expression is upregulated by UL138, which has a dominant effect. Other virally encoded functions may downregulate TNFR1 expression (Montag et al., 2011). The WCL pattern of expression of UL138 is shown overlying both PM and WCL data.

Red diamonds – 12h after infection with irradiated HCMV.
